# Design of a spermine-β-cyclodextrin functionalized gelatin nanoaggregate hydrogel for nose-to-brain delivery of donepezil

**DOI:** 10.1039/d6ra01040g

**Published:** 2026-09-01

**Authors:** Zaynab Mokhtari, Sedigheh Hashemnia, Reza Heidari, Mohammad Hossein Mokhtari

**Affiliations:** a Department of Chemistry, Faculty of Nano and Bio Science and Technology, Persian Gulf University Bushehr 75169 Iran shashemnia@pgu.ac.ir; b Department of Chemical Industry, Technical and Vocational University (TVU) Bushehr Iran; c Pharmaceutical Sciences Research Center, Shiraz University of Medical Sciences Shiraz Iran; d Department of Medicinal Chemistry, School of Pharmacy, Mashhad University of Medical Sciences Mashhad Iran

## Abstract

Rapid mucociliary clearance and limited permeability across biological barriers remain major challenges for efficient nose-to-brain delivery of donepezil (DNP). Herein, we report a rationally engineered biodegradable gelatin hydrogel in which chemically conjugated spermine-β-cyclodextrin (SPM-β-CD) nanoaggregates were integrated to enhance intranasal nose-to-brain delivery of donepezil. Gelatin served as a biodegradable hydrogel matrix, while β-cyclodextrin provided host–guest inclusion sites for efficient donepezil encapsulation and spermine promoted supramolecular nanoaggregate formation within the polymer network. The hydrogel was synthesized through EDC/NHS-mediated crosslinking, generating a highly interconnected hierarchical architecture that regulated swelling behavior, structural stability, hydrolytic biodegradation, and diffusion-controlled drug release. Consequently, the optimized formulation (DNP/F-GHyd2) exhibited sustained DNP release together with pronounced free radical scavenging activity associated with the functional biomaterial components. *In vivo* pharmacokinetic evaluation in Sprague-Dawley rats showed significantly enhanced brain accumulation of DNP following intranasal administration of DNP/F-GHyd2 compared with free DNP solution, with brain maximum concentration (*C*_max_) values of 0.059 ± 0.006 µg mL^−1^ and 0.024 ± 0.005 µg mL^−1^, respectively. Histopathological assessment further confirmed the biocompatibility of the developed hydrogel system with no detectable tissue alterations in brain sections. Collectively, these findings establish a clear structure–property–function relationship, demonstrating that chemically engineered SPM-β-CD nanoaggregates function as molecular regulators of gelatin hydrogel architecture. To the best of our knowledge, this study is the first to report a chemically engineered SPM-β-CD nanoaggregate gelatin hydrogel that utilizes supramolecular nanoaggregate engineering for sustained intranasal delivery of donepezil.

## Abstract 2

Rapid mucociliary clearance and limited permeability across biological barriers remain major challenges for efficient nose-to-brain delivery of donepezil (DNP).

## Graphical abstract

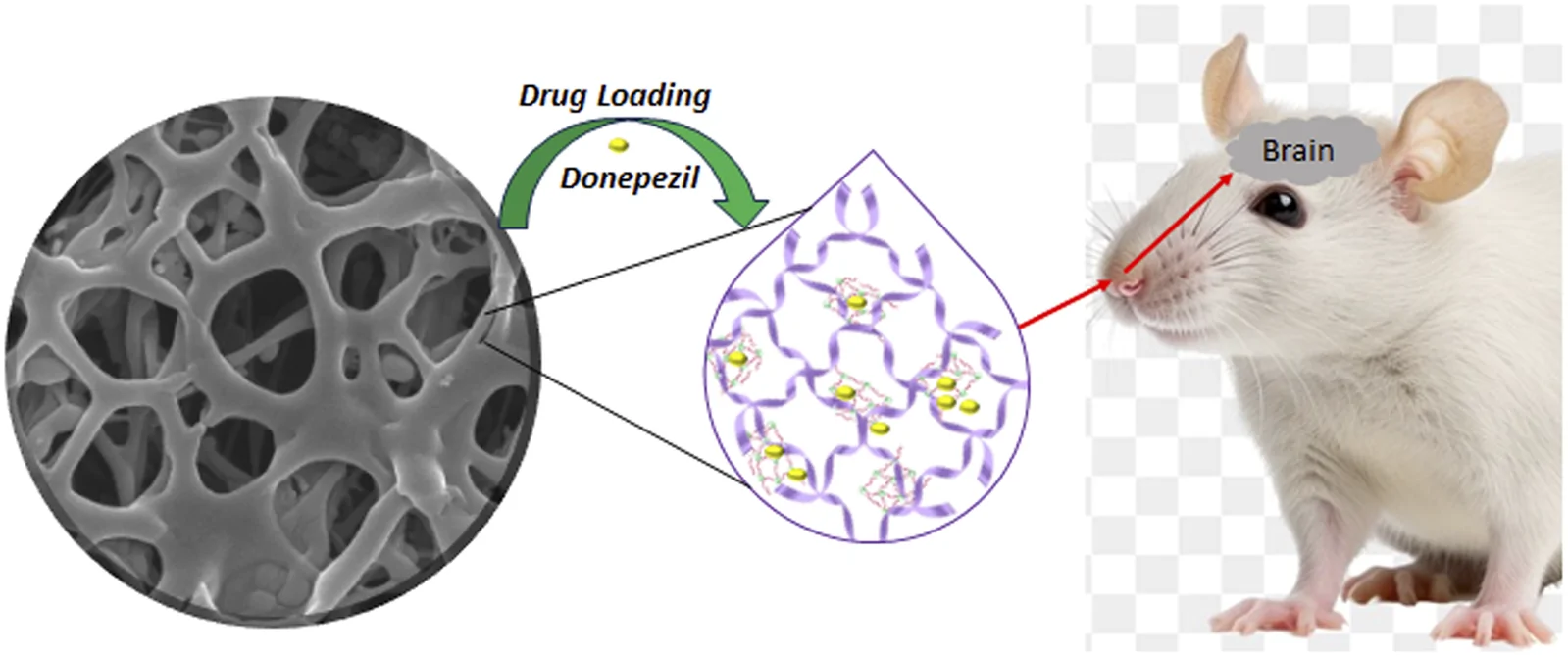

## 1. Introduction

Age-related neurodegenerative disorders, particularly Alzheimer's disease (AD), are rapidly increasing worldwide and represent a major global health challenge. According to the WHO, approximately 47.5 million people were affected by dementia in 2017,^1^ with AD accounting for the majority of cases. AD is a progressive neurodegenerative disorder characterized by synaptic dysfunction, cholinergic system impairment, and the accumulation of neurofibrillary tangles and β-amyloid plaques in the brain.^2^ The disruption of cholinergic neurotransmission leads to cognitive decline, memory loss, and behavioral impairments that significantly affect quality of life. Current pharmacological management of AD primarily relies on cholinesterase inhibitors, which aim to enhance acetylcholine availability at synaptic junctions by preventing its enzymatic degradation. Among these agents, Donepezil hydrochloride (DNP) is widely prescribed across mild to severe stages of AD due to its relatively favorable safety profile and clinical efficacy. DNP acts as a reversible acetylcholinesterase inhibitor, thereby increasing cerebral ACh levels and alleviating cognitive symptoms.^3^ However, conventional DNP administration is often associated with limited brain bioavailability, systemic side effects, and insufficient targeting efficiency. Consequently, numerous nanocarrier-based delivery systems, including polymeric nanoparticles, lipid nanocarriers, nanoemulsions, and hydrogel-based formulations, have been explored to improve the brain delivery of DNP. Despite these advances, achieving efficient brain targeting while simultaneously ensuring prolonged nasal residence, structural stability, controlled drug release, and biocompatibility remains a major challenge.^2–7^ Although DNP is widely available as oral capsules or tablets, its clinical use is frequently associated with adverse effects, including gastrointestinal discomfort such as diarrhea. Moreover, as dementia progresses, dysphagia and swallowing problems become increasingly prevalent, further limiting the suitability of oral administration. These challenges highlight the need for alternative, non-oral delivery strategies to improve patient compliance and therapeutic efficacy. Among the various routes of administration, the intranasal route has attracted considerable attention due to its potential for direct drug transport to the brain. Following intranasal administration, drug molecules can reach the olfactory bulb and trigeminal regions through the olfactory and trigeminal pathways, subsequently entering the brain *via* diffusion-based processes. This direct nose-to-brain transport enables therapeutic agents to bypass the blood–brain barrier (BBB), a major physiological obstacle that restricts central nervous system delivery of many newly developed drugs.^1^ Other advantages of nasal administration include the avoidance of hepatic first-pass metabolism, the possibility of incorporating various absorption enhancers to improve drug bioavailability, and enhanced permeability compared to the gastrointestinal tract due to the absence of pancreatic and gastric enzymes.^8,9^

About 20–50% of nasal epithelial cells possess motile cilia (approximately 200–300 cilia per cell), which are primarily responsible for mucociliary clearance. This clearance mechanism represents a critical physiological defense system that significantly limits the efficiency of intranasal drug administration. Rapid mucociliary clearance significantly limits the residence time of intranasal formulations on the nasal mucosa, thereby reducing drug absorption and therapeutic efficiency.^10^ To address the rapid mucociliary clearance associated with intranasal administration, hydrogel-based nanosystems have emerged as versatile biomaterial platforms. Hydrogels are three-dimensional (3D) polymeric networks formed through physical or chemical cross-linking, capable of absorbing large amounts of water while maintaining structural integrity. Their hydrophilic nature originates from functional moieties such as hydroxyl, amide, and sulfonic groups distributed along the polymer backbone, which also contribute to network formation. From a drug delivery perspective, hydrogels offer several advantageous features, including the ability to form stable formulations under physiological conditions, tunable network architectures that enable high drug loading, and controlled release profiles at the target site. Moreover, the presence of reactive functional groups allows for chemical modification and ligand-mediated functionalization, facilitating site-specific interactions and improved targeting efficiency. Owing to these properties, hydrogel-based biomaterials have demonstrated considerable potential for application across multiple administration routes, including intranasal, oral, transdermal, buccal, and pulmonary delivery systems.^11^ Nevertheless, the performance of hydrogel-based carriers is dictated not only by the choice of constituent biomaterials but also by the molecular organization of the hydrogel network, which determines water diffusion, structural integrity, degradation behavior, and drug transport. Therefore, rational engineering of hydrogel architecture has become an emerging strategy for improving intranasal drug delivery.

Gelatin, a natural and water-soluble polymer, is widely employed in drug delivery applications owing to its favourable biocompatibility and versatility. It helps to maintain moisture in the nasal cavity, can form a barrier against irritants or pathogens, and supports tissue repair processes, thereby potentially reducing local inflammatory responses.^8^ Among naturally occurring polyamines, spermine has attracted increasing attention as a multifunctional biomolecule because of its antioxidant activity, membrane-interaction capability, and potential to improve transport across biological barriers.^12–15^ Several intranasal donepezil delivery systems, including chitosan hydrogels, pluronic-based gels, and hyaluronic acid-coated nanocarriers, have been previously reported to improve nasal permeation and brain targeting.^16–18^

Despite these advances, previously reported intranasal donepezil delivery systems have primarily focused on optimizing formulation composition or carrier type, whereas comparatively little attention has been paid to molecular engineering of hydrogel architecture as a means of simultaneously regulating physicochemical properties and biological performance. In particular, no previous study has reported a chemically engineered gelatin hydrogel incorporating spermine-functionalized β-cyclodextrin (SPM-β-CD) nanoaggregates to establish a direct structure–property–function relationship for intranasal donepezil delivery.

Because the nasal mucosa is highly sensitive to chemical and mechanical irritation, the selection of biocompatible and non-irritating biomaterials is essential for intranasal delivery systems. Repeated administration of nasal therapeutics may induce mucosal irritation or lead to local tolerance, ultimately compromising therapeutic efficacy. In this context, the proposed nasal formulation integrating gelatin, spermine and β-cyclodextrin (β-CD) may offer additional advantages beyond drug delivery performance. Spermine and gelatin may additionally contribute to antioxidant and anti-inflammatory effects while improving mucosal compatibility.^19–21^ Based on the above considerations, the incorporation of spermine as a functional component of the intranasal carrier system is anticipated to enhance the performance of the hydrogel, particularly in light of its antioxidant activity within the brain.

An important advantage of the proposed spermine-β-cyclodextrin-polyamine (SPM-β-CD) nanoaggregate hydrogel system is the use of inherently biocompatible and biodegradable biomaterials. β-CD can undergo enzymatic degradation, while spermine and gelatin are naturally metabolizable biomolecules. In addition, the hydrogel network was fabricated without the use of toxic crosslinking agents, further supporting its biomedical applicability. These characteristics are particularly advantageous for intranasal administration, where biodegradable carriers are desirable to minimize long-term mucosal accumulation during repeated dosing. Previous studies have demonstrated that polyamine-functionalized biomaterials can enhance transport across biological barriers, including the BBB and blood–nerve barrier (BNB), supporting the rationale for incorporating spermine into the present nose-to-brain delivery platform.^22–24^

In this study, we report, to the best of our knowledge, the first chemically engineered gelatin hydrogel incorporating spermine-functionalized β-cyclodextrin (SPM–β-CD) nanoaggregates for intranasal delivery of donepezil. Rather than simply combining gelatin, β-cyclodextrin, and spermine as formulation components, the proposed molecular design generates a hierarchical supramolecular nanoaggregate architecture that regulates swelling, structural stability, hydrolytic biodegradation, thermal behavior, and drug-release kinetics. Through this molecular organization, a clear structure–property–function relationship is established, linking supramolecular network architecture with enhanced brain targeting, sustained drug release, improved pharmacokinetic performance, antioxidant activity, and excellent *in vivo* biocompatibility. These findings introduce a rational molecular engineering strategy for designing next-generation biodegradable hydrogel platforms for nose-to-brain drug delivery.

## 2. Materials

All chemicals were of analytical grade and purchased from Aldrich or Merck company except donepezil hydrochloride, which was received as a gift from Nikan Exir Bakhtar pharmaceutical company.

## 3. Methods

### 3.1. Synthesis of carboxymethyl β-cyclodextrin (CM-β-CD)

**Scheme 1:**
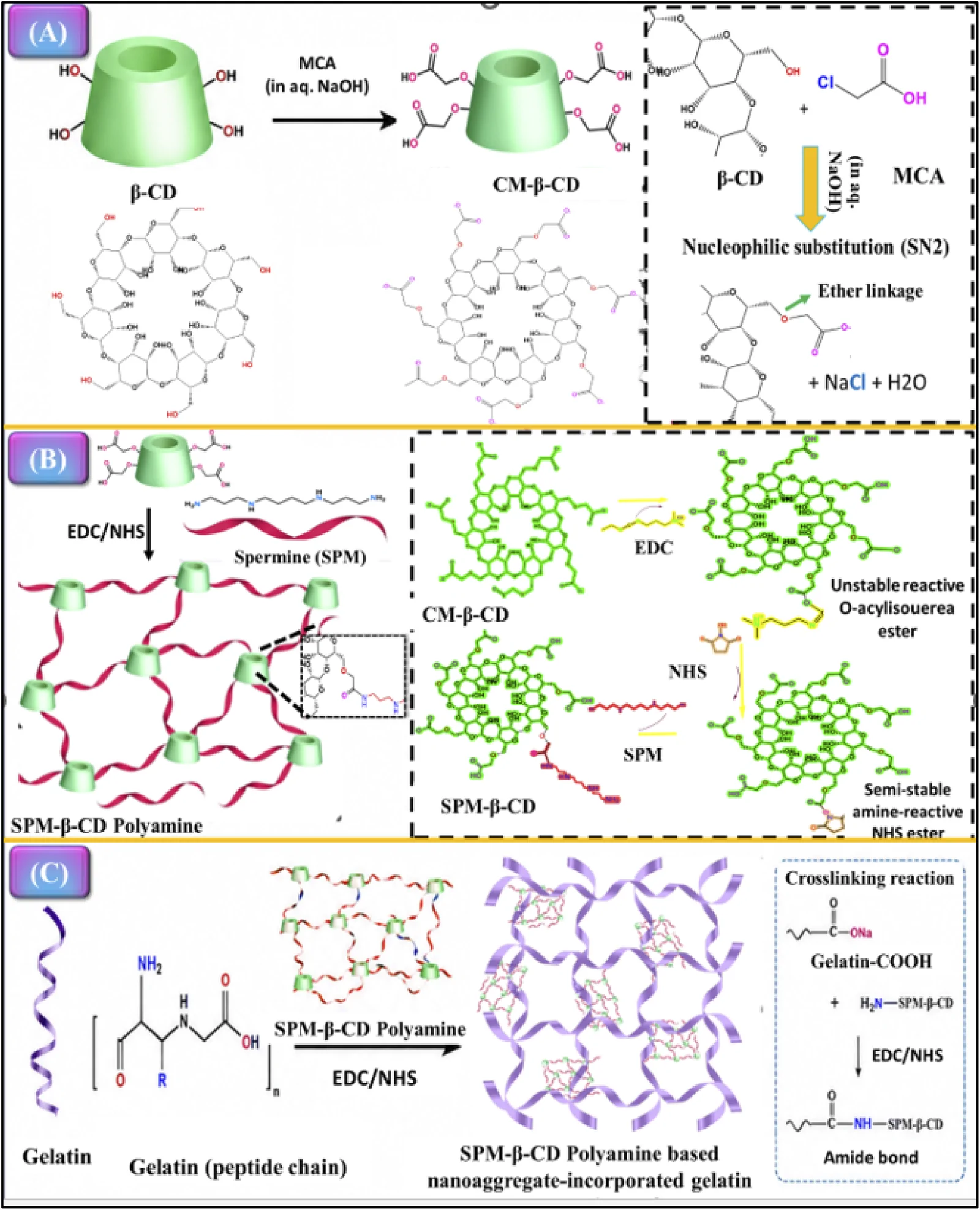
The illustrations of (A) CM-β-CD, (B) SPM-β-CD polyamine and (C) SPM-β-CD-based nanoaggregate-incorporated gelatin hydrogel (F-GHyd) preparation.

The hydrogel containing nanopolymeric aggregates was prepared in several steps. In the first step, carboxymethyl β-cyclodextrin (CM-β-CD) was prepared. For this purpose, β-CD (2.0 g) and monochloroacetic acid (MCA) (2.0 g) were dissolved separately in an aqueous solution (50 mL) containing 5% W/V NaOH. The mixture was then stirred magnetically for 30 min to dissolve completely and make for the deprotonation of the hydroxyl and carboxyl groups. The solutions were combined and then stirred without heating for 24 h. The obtained solution was neutralized to pH 7.0 using a 3.0 M solution of HCl. Then, the resulting solution was concentrated to about one-third of its previous volume, and the precipitation was obtained by adding a cold ethanol solution (70% °C). After settling the precipitate and pouring off the supernatant solution, the precipitates were washed with cold ethanol solution (70% v/v) again and dried at 70 °C, under vacuum for 5 h (Scheme 1A). The resulting precipitate (CM-β-CD) was characterized using FT-IR and 1H NMR (SI, Fig. S1) and stored at 5 °C for further use.^25^

### 3.2. Preparation of spermine-β-CD polyamine complex (SPM-β-CD)

The SPM-β-CD polyamine was attained by reacting spermine (SPM) with CM-β-CD *via* 1-ethyl-3-(3-dimethylaminopropyl)carbodiimide/*N*-hydroxysuccinimide (EDC/NHS) coupling agents (Scheme 1B). The synthesized CM-β-CD (0.45 g) was dissolved in a 0.1 M phosphate-buffered solution (PBS) (25 mL, pH 5.0) by stirring. Then EDC (0.21 g) and NHS (0.21 g) were added to the solution. After 30 min, a buffered solution (25 mL, 0.1 M, pH 5.0) containing SPM (0.14 g) was added to the obtained solution and stirred for 24 h at room temperature. The mixture was concentrated to obtain a viscous fluid, and then the precipitate was collected by the addition of a cold ethanol (70% v/v). In order to purify the product (SPM-β-CD), a cold ethanol solution (70% v/v) was added to it again and after overflowing of the supernatant solution, the precipitate was collected and dried under vacuum for 24 h.^25,26^

### 3.3. Formulation of gelatin hydrogels containing SPM-β-CD (F-GHyd)

A series of gelatin hydrogels containing SPM-β-CD polyamine were made by changing the amounts of SPM-β-CD to gelatin powder in the reaction mixture. In general, the procedure for the synthesis of a gelatin hydrogel (GHyd) containing SPM-β-CD polyamine consisted of a two-step reaction (Scheme 1B). Initially, the gelatin hydrogel (GHyd) synthesized. In the second step, the conjugation of SPM-β-CD to the GHyd was done utilizing a typical carbodiimide ester-mediated coupling reaction. For the first step, six different aqueous solutions containing gelatin powder (5.2 g, 300 mL) were prepared and stirred magnetically at 70 °C for 7 h. By adding an HCl solution (3.0 M), the pH value of the obtained solution was adjusted to 3.0. Then, EDC (0.2 g) and NHS (0.2 g) were added to the solution. In the second step, different amounts of SPM-β-CD (0.5, 2.0, 5.0, and 10.0 g) were thoroughly dissolved in 100 mL distilled water using a stirrer, and the pH of these solutions was also adjusted to 3.0. Each SPM-β-CD solution was then added dropwise with a syringe (1.0 mL) to the prepared gelatin hydrogel solution. Subsequently, the conjugation was completed at 40 °C for 24 h, under continuous stirring. The hydrogel purification was done using a dialysis membrane (50 kDa) with deionized water until the solution conductivity was close to that of distilled water. The purified hydrogel dispersion was solvated in an ethanol (70% v/v) solution, followed by applying a vacuum rotary at 60 °C to evaporate the solvent (Scheme 1).

The hydrogels were dispersed again in the ethanol solution (70% v/v). The desolvation/drying and re-dispersion process was done consecutively three times to produce gelatin hydrogels containing nanopolymeric aggregates of SPM-β-CD (F-GHyd) with a porous network, and the hydrogel polymers named F-GHyd1, F-GHyd2, F-GHyd3, and F-GHyd4 based on the addition of the various amounts of SPM-β-CD (0.5, 2.0, 5.0 and 10.0 g) to the gelatin solution (Scheme 1C). The resulting hydrogel was re-dispersed in water *via* ultrasonication (10 min), then autoclaved at 120 °C for 20 min, afterward lyophilized employing a vacuum freeze dryer, and kept at 4 °C for later use. As mentioned before, three other hydrogel polymers, gelatin hydrogel (GHyd) and β-CD-gelatin hydrogel (CDGHyd), and spermine-gelatin hydrogel (SPMGHyd) were fabricated. The former was prepared from gelatin powder in the absence of another compound, and, in the latter cases, CM-β-CD and spermine were added instead of SPM-β-CD, respectively.^26,27^

### 3.4. Choosing the best hydrogel formulation and optimization of the formulation percentage

Employing the following formula (eqn (1)), the encapsulation efficiency (%EE) of DNP in hydrogel formulations was estimated.^28,29^1
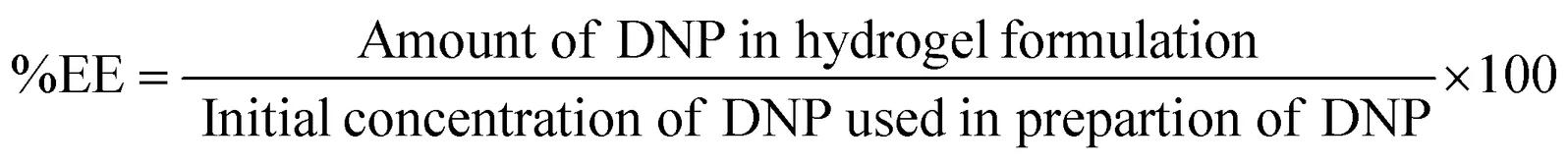


The amount of the loaded DNP in the hydrogel formulations was measured utilizing an ultraviolet-visible (UV/vis) spectrometer (Analytic Jena, SPECORD 250, Germany). The DNP stock solution (1.5 mg mL^−1^) in PBS was stored in Eppendorf tubes at −20 °C. Afterward, different working standard solutions of DNP were made from the stock solution. The absorbance diagrams of standard samples of DNP (0.55 to 71.00 µg mL^−1^ in PBS) were measured in the wavelength range of 200–380 nm. A calibration curve was obtained using the absorbance at 272 nm at varying concentrations of DNP (Fig. S2). The DNP-loaded hydrogel formulations were quantified based on the obtained calibration graph. The purpose of using different percentages of each formulation was to obtain the optimum amount of hydrogel based on the higher entrapment efficiency of DNP.

### 3.5. The behaviour of swelling

At first, a specific weight (*W*_d_) of the dried hydrogels was immersed in PBS (pH 7.40) at room temperature to hydrate. Then, the hydrated hydrogels were dried softly on filter paper to remove any excess water on the surface and then weighed (*W*_s_). The swelling capacity of hydrogels represents the hydrogel capability to absorb water and is calculated using eqn (2):^30,31^2
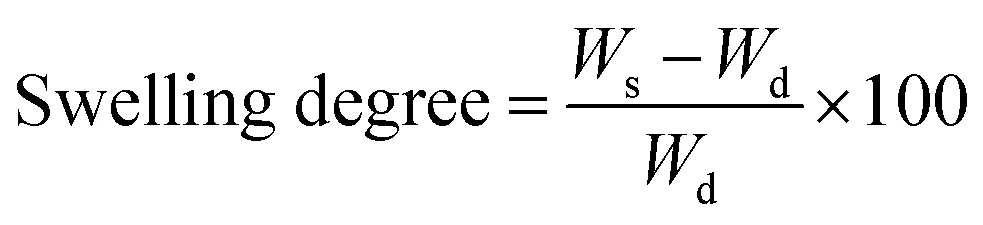


### 3.6. *In vitro* hydrolytic degradation

The *in vitro* hydrolytic degradation behavior of the prepared hydrogels was evaluated in PBS (pH 7.4). Freeze-dried hydrogel samples (10.0 ± 2.0 mg) were weighed to obtain the initial dry weight (*W*_0_) and immersed in 5 mL of PBS. The samples were incubated at 37 °C under static conditions. The hydrogel samples were collected after 1, 3, and 5 days, respectively. At each predetermined time point, the corresponding hydrogel sample was removed from the degradation medium, gently rinsed with deionized water to remove residual buffer salts, and dried in an oven at 60 °C to constant weight. The remaining dry weight (*W*_*t*_) was then recorded. Fresh PBS was used for each degradation period. The degradation percentage was calculated according to eqn (3).3
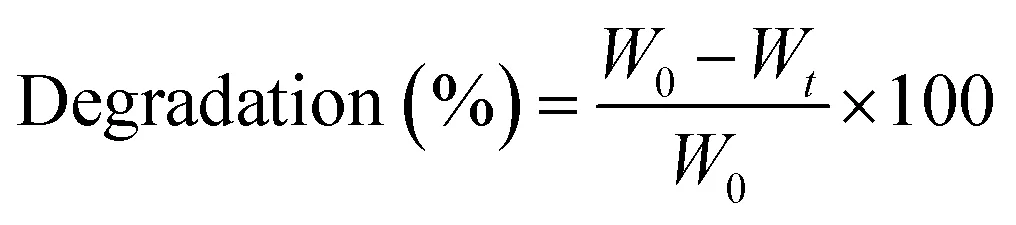
where *W*_0_ and *W*_*t*_ represent the initial dry weight and the remaining dry weight after hydrolytic degradation, respectively.

### 3.7. *In vitro* release experiments

The release of DNP from different hydrogel formulations was measured using the dialysis bag technique (50 kDa). The *in vitro* drug release profile of the prepared hydrogels was carried out using 50 mL of the PBS solution at 7.4 pH (at 25 °C). Then, 1 mL of solution around the dialysis bag was removed at the selected time intervals and replaced by 1 mL of the PBS solution at 7.4 pH. Then, the cumulative percentage of DNP was measured at 272 nm, applying the UV-visible spectrophotometer. *In vitro* release data from formulations F-GHyd1, F-GHyd2, F-GHyd3, and F-GHyd4 were integrated into kinetic models, including zero order, first order, Higuchi and Korsmeyer–Peppas models. The mechanism of drug release was characterized by the Korsmeyer–Peppas model, considering the value of *n*.^6^

### 3.8. *In vivo* experiment

The *in vivo* investigations of the optimized DNP/F-GHyd2 and free DNP solution were conducted on male Sprague–Dawley rats (weighing 250–300 g) acquired from the Animal House, Shiraz University of Medical Sciences (Iran). The rats were housed in standard cages and were provided with a standard laboratory diet and water ad libitum. A total of 30 male Sprague–Dawley rats were used in this study. Following acclimatization, the animals were randomly allocated into two experimental groups (free DNP solution and DNP/F-GHyd2; *n* = 15 per group). Prior to intranasal administration, the animals were deeply anesthetized with thiopental sodium (100 mg kg^−1^, i.p.). The DNP/F-GHyd2 formulation (containing 0.5 mg mL^−1^ of DNP) and free DNP (0.5 mg mL^−1^) solutions were administered to the rats through the intranasal route, and the brain and blood samples were collected at time intervals 1, 2, 4, 6 and 12 h after administration. To perform IN administration, 20 µL of DNP solution or DNP/F-GHyd2 formulation was poured into each nostril with a sampler. The blood (1.5 mL) samples were drawn into polyethylene micro tubes (capacity of 2 mL) containing a sodium citrate 4% w/v solution (50 µL) as an anticoagulant. The blood samples were centrifuged (1000 rpm, 10 min, 112×*g*) to separate the plasma and stored at −21 °C up to the time of measurements. The brain samples (250 mg) were put in screw-cap glass tubes. A solution of sodium chloride (2.5 mL, 0.90% w/v) was added to the brain samples and then homogenized. After the IN administration of the free DNP solution and DNP/F-GHyd2, the DNP levels were measured at the time points 1, 2, 4, 6 and 12 h. For histopathological evaluation, tissue samples were fixed with 10% buffered formalin. Sections were obtained from the middle region of the brain and deparaffinized using ethanol and xylene to remove embedded paraffin from the brain tissues. Histopathological assessment was performed by an experienced veterinary pathologist who was blinded to the treatment groups to minimize observer bias. Finally, samples were stained with hematoxylin and eosin to monitor any sign of the toxicity of investigated nasal carrier. Pharmacokinetic parameters were analyzed using non-compartmental analysis with the help of software (PK Functions for Microsoft Excel).

### 3.9. Evaluation of the amount of drug in the animal samples by the HPLC method

The brain tissue and plasma samples (200 µL) (or with a standard DNP solution) were homogenized in 1% acetic acid (2 mL) separately. Then, using centrifugation, the suspended particles were removed. 20 µL of the clear supernatant was injected into the YOUNG LIN system using an HPLC injector to analyze the DNP content using the HPLC technique. The chromatographic analysis was carried out using a C18 column (250 mm × 4.6 mm, with 5 µm particle size). The utilized mobile phase was A: (40%v): B (50%v): C (10%v) (A: phosphate buffer (0.02 M pH 7.4), B: methanol, C: acetonitrile). The 1 mL min^−1^ flow rate was adjusted for the mobile phase. Detection of DNP was done at 280 nm. The concentration–time plots were obtained for DNP. Software package Kinetica v 4.4 was applied to carry out the noncompartmental pharmacokinetic analysis. All data were represented as mean ± SD between experiments. Different pharmacokinetic parameters such as *C*_max_ (maximum drug concentration), *T*_max_ (time required to reach *C*_max_), AUC0-12 (area under the curve), AUMC0-12 (area-under-the-moment-curve), elimination rate constant (Ke), and mean residence time (MRT) were computed.^5,6^

### 3.10. Stability study

Based on the results, the DNP/F-GHyd2 formulation represented the best results among different hydrogels; therefore, we considered DNP/F-GHyd2 to explore long-term stability. The developed DNP/F-GHyd2 was kept at 5 °C for three months to check out the time stability based on mean size (using DLS in different periods: two weeks, one month, and three months), radical scavenging based on DPPH assay and *in vitro* drug release evaluation after 3 months.

### 3.11. Radical scavenging activity (DPPH assay)

The radical scavenging analysis was done employing 2, 2-diphenyl-1 picrylhydrazyl (DPPH) as stable radicals. The DPPH radical solutions show a violet color and a strong absorption at about 517 nm as a result of its odd electron. Then, the color vanishes in the presence of electron-donating compounds. Therefore, 50 µL of samples (2.5 mg mL^−1^of DNP/F-GHyd2 and 0.1 mg mL^−1^of free DNP) was added to the DPPH ethanol solution (2 mL, 0.16 mM) and incubated in the dark at room temperature for 30 min. Then, the absorbance was measured at 517 nm, and the scavenging activities of the samples were calculated by the following equation (eqn (4)):4
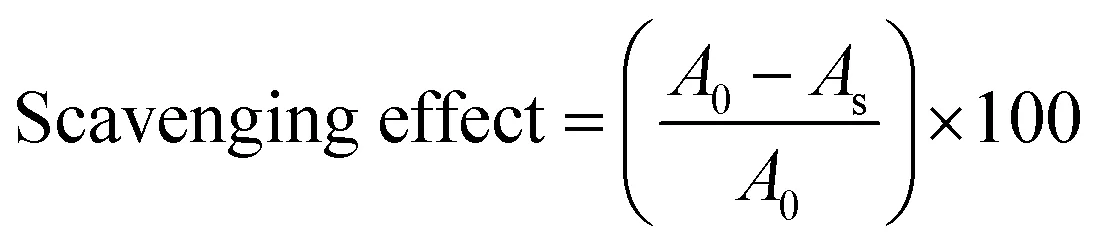
In this equation, *A*_0_ is the absorbance of the blank DPPH solution, and *A*_s_ represents the absorbance of the DPPH solution in the presence of the samples.^32^

### 3.12. Statistical analysis

All experiments were performed in triplicate (*n* = 3), and data are presented as mean ± standard deviation (SD). Comparisons among multiple groups were performed using one-way analysis of variance (ANOVA) followed by Tukey's honestly significant difference (HSD) post hoc test. Pairwise comparisons of pharmacokinetic parameters were performed using an unpaired two-tailed Student's *t*-test. Differences were considered statistically significant at *P* < 0.05.

## 4. Results

### 4.1. Preparation and characterization of the synthesized SPM-β-CD

**Fig. 1:**
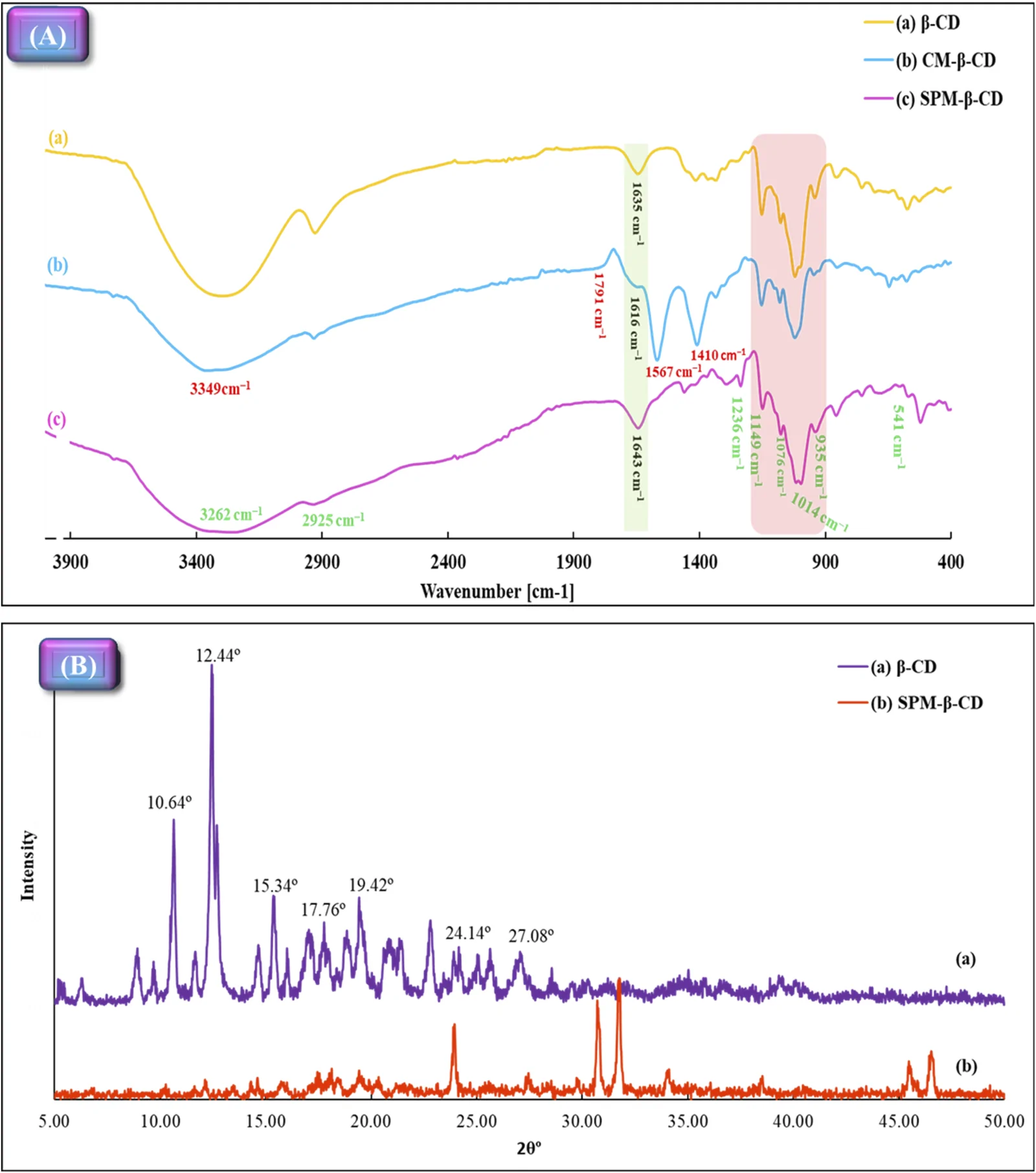
(A) FT-IR spectra of (a) β-CD, (b) CM-β-CD, (c) SPM-β-CD, (B) XRD patterns of the (a) β-CD and (b) SPM-β-CD.

The present study proposes a simple and two-step chemical modification method to produce the new biopolymer F-GHyd. In the first step, CM-β-CD was aminated by spermine (SPM) to obtain a polyamine compound named SPM-β-CD (Scheme 1A). To assess the synthesis of SPM-β-CD from CM-β-CD, FTIR, XRD, and SEM techniques were applied. As shown in Fig. 1A, the FT-IR peaks around 2925, 1149, 1076, 1014, and 935 cm^−1^ are related to the main β-CD skeletal structure. These are assigned to the aliphatic C–H stretches, C–C/C–O and C–O–C stretching vibrations, and 1, 4-glycosidic structural skeleton of β-CD. These bands are also revealed in the CM-β-CD and SPM-β-CD spectra and show the maintenance of the β-CD structure. The appearance of a new peak in the region of 1791 cm^−1^, and the peaks in the regions of 1567 and 1410 cm^−1^ in the CM- β-CD spectrum (Fig. 1A (curve b)) are connected to the stretching vibration of carbonyl groups and the symmetric and asymmetric stretch of carboxyl groups, respectively, which confirms the carboxymethylation of β-CD. Furthermore, the appearance of a new peak at 1236 cm^−1^ in the SPM-β-CD spectrum (curve c) can be assigned to the C–N stretching of amide groups, owing to the coupling of the SPM amine groups with the carboxylate groups of CM-β-CD. The characteristic OH peak at 3349 cm^−1^, in the spectrum of CM- β-CD, was shifted to 3262 cm^−1^ in the spectrum of SPM-β-CD because of the overlap between –OH stretching vibration and the –NH_2_ stretching band. The shift in the C=O band to 1643 cm^−1^ upon forming the SPM-β-CD spectrum and the observed band at 541 cm^−1^ (related to the NH amide band) confirm the formation of amide groups.^33,34^

**Fig. 2:**
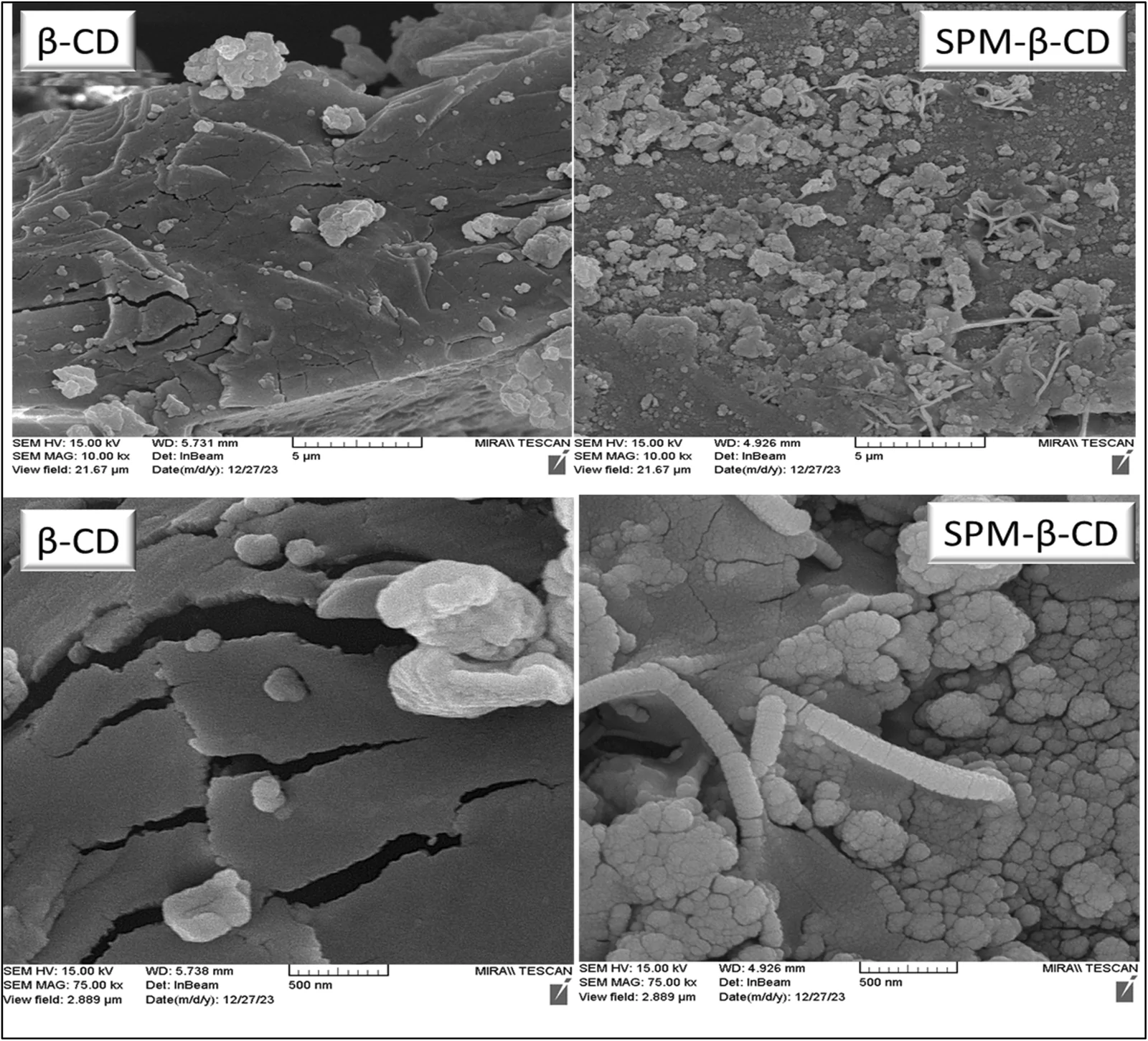
FESEM images of β-CD and SPM- β-CD with two different magnifications for each of them (5 µm and 500 nm).

The SPM-β-CD polyamine complex production is a critical step in the design of the proposed hydrogel carrier, so precise characterization of this step is important. Monitoring the variations of the β-CD crystal structure after amination by SPM implies the incorporation of SPM and β-CD. The XRD pattern of β-CD (Fig. 1B) represents its highly crystalline structure, identified by several intense and distinctive peaks at 2*θ* = 10.64, 12.44, 17.76, 19.42, 24.14 and 27.08°. With the covalent binding of SPM to the oxidized CD structure ((Fig. 1B, Curve b), the sharpness of the crystalline peaks decreased because of the involvement of the hydroxyl groups of β-CD interacting with SPM, and the SPM-β-CD polyamine structure became nearly amorphous.^35,36^ Also, SEM images were employed to investigate the morphology of the synthesized SPM-β-CD polyamine. β-CD displays a rough and cracked morphology that could be assigned to the structure crystallinity (Fig. 2). After the modification of the oxidized β-CD by SPM (Fig. 2), the amorphous aggregates comprised of clusters with separate particles were observed, demonstrating the covalent attachment of the β-CD to the SPM.

The difference between the morphologies can be evidence of the SPM-β-CD polyamine formation.^37,38^ In order to design SPM-β-CD-based nanoaggregate-incorporated gelatin hydrogels (F-GHyd), in the second step, the chemistry of EDC/NHS crosslinker was applied (Scheme 1B). The incorporation of the SPM-β-CD polyamine in the gelatin hydrogel was then evaluated by FT-IR spectroscopy.

**Fig. 3:**
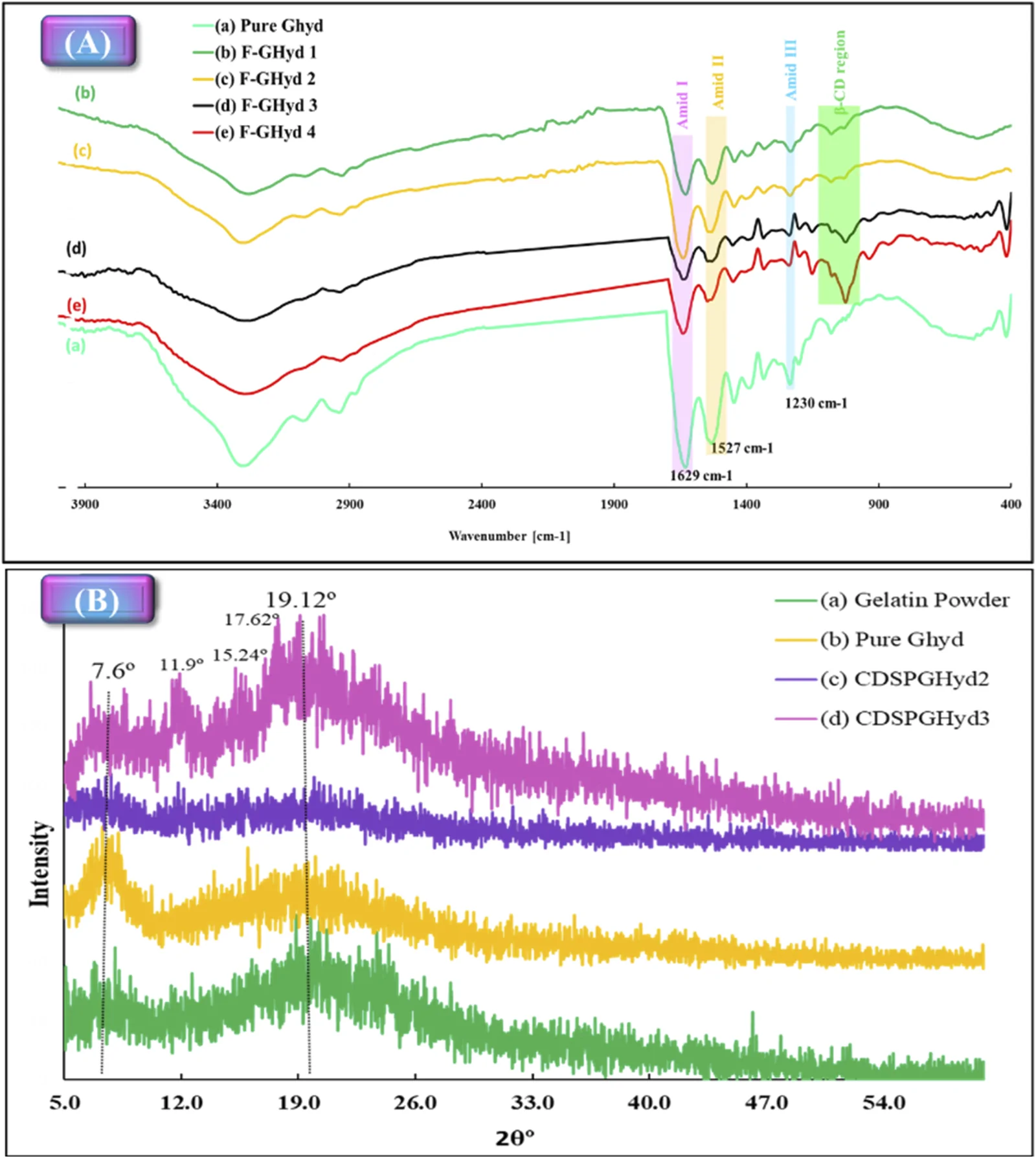
(A) FTIR spectra of (a) GHyd and (b) F-GHyd1, (c) F-GHyd2, (d) F-GHyd3, and (e) F-GHyd4, (B) XRD patterns of the (a) gelatin powder, (b) GHyd, (c) F-GHyd2 and (d) F-GHyd3.

Fig. 3A represents the FTIR spectra of the pure GHyd and the modified gelatin hydrogel by four different amounts of SPM-β-CD (as F-GHyd1, F-GHyd2, F-GHyd3 and F-GHyd4). All spectra show the characteristic adsorption bands of amide I (1629 cm^−1^) and II (1527 cm^−1^). The amide I band is most extensively employed for studies of protein secondary structure and is a characteristic of the C=O stretching vibration of the amide group. In Amide II, N–H in-plane bending and C–N stretching vibration created an absorption band at the 1527 cm^−1^ (Fig. 1A spectra). Amide III C–N symmetry vibration resulted in the absorption band at about 1230 cm^−1^. It can be observed from the modified hydrogels spectrum, compared to the unmodified pure GHyd, that the new peaks were produced in the 1149 to 935 cm^−1^ region, which was related to the presence of the β-CD skeleton and were intensified by increasing the SPM-β-CD percentage in the modified hydrogels (F-GHyd). Therefore, the presence of the characteristic bands of both gelatin and SPM-β-CD in the modified hydrogels confirms the incorporation of the SPM-β-CD with pure Ghyd (Fig. 3A).^39,40^ The physical nature of the developed hydrogels was observed using XRD patterns. The XRD patterns of gelatin powder, pure GHyd, and the modified hydrogels (F-GHyd) are shown in Fig. 3B. The gelatin powder as raw material (Fig. 3B Curve a) shows two diffraction peaks at 2*θ* = 7.6° and 19.12°. Compared to the gelatin powder, the peak intensity at 2*θ* = 7.6° (characteristic of the regular triple helical crystalline structure) for the developed pure GHyd is increased, directly revealing the ordered triple helix structure. α-helix and the random coil are the major conformations that explain the gelatin structure.

**Fig. 4:**
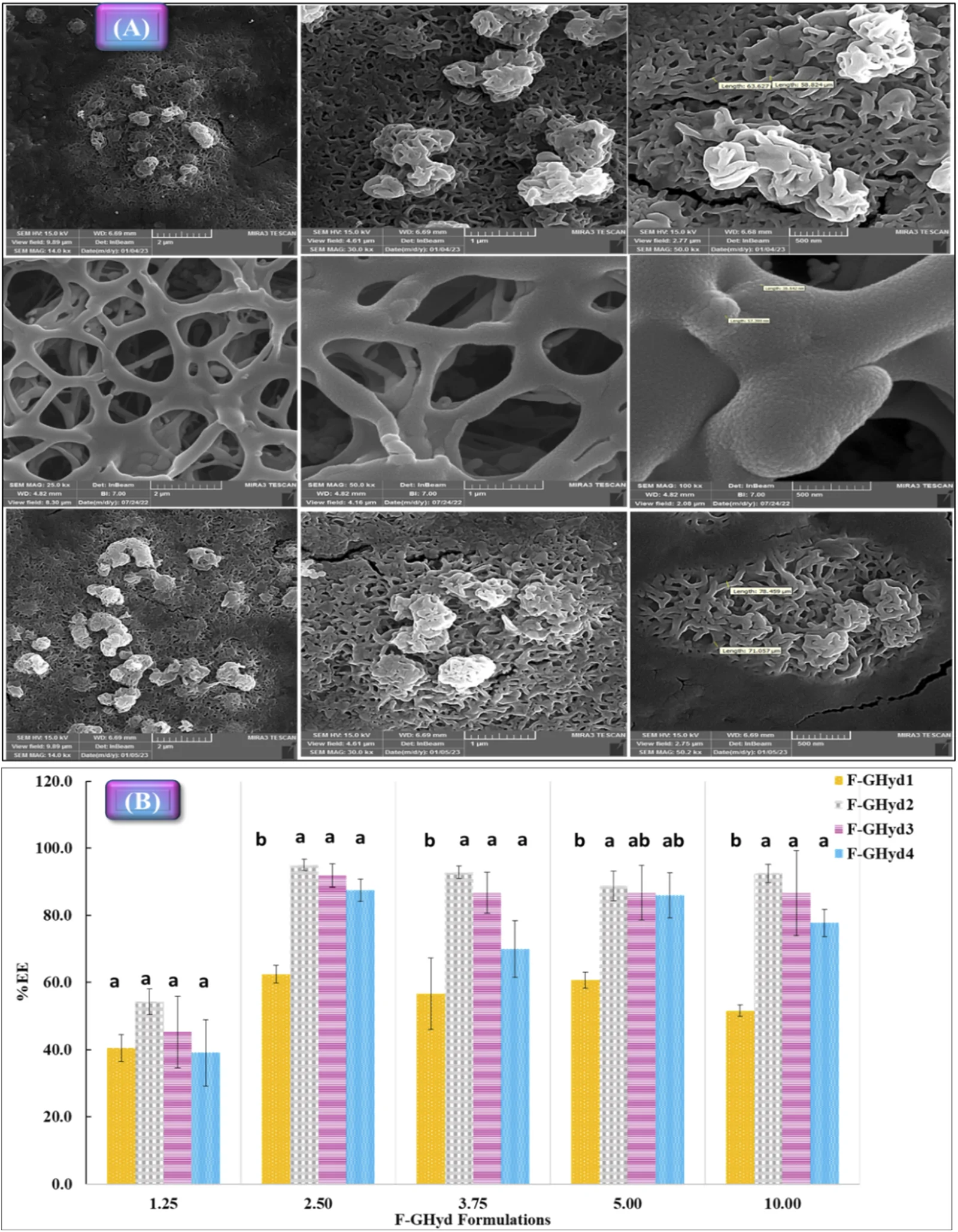
(A) SEM images of (a) GHyd, (b) F-GHyd2, (c) F-GHyd3, (three different magnifications are shown for each sample (2 µm, 1 µm, and 500 nm), (B) encapsulation efficiency percentage (EE%) for different formulations (F-GHyd1-4) with various concentrations of SPM-β-CD. (Mean ± SD, *n* = 3), different lowercase letters indicate statistically significant differences among formulations according to one-way ANOVA followed by Tukey's multiple comparison test (*P* < 0.05).

Hydrogen bonds are most responsible for the stability of the α-helix conformation and triple-helix structures in gelatin. Based on reports, water molecules forming hydrogen bonds between adjacent gelatin chains like a bridging factor appear to have a crucial effect on the triple helix stability. In this study, the complex of SPM-β-CD is attached to the gelatin functional groups (C=O and N–H) *via* covalent attachment. The hydrophilic groups such as OH, CO, NH_2_, and NH, which exist in the SPM-β-CD polyamine structure, tend to interact with water molecules *via* hydrogen bonds. As a result, they prevent the water molecules from interacting with gelatin chains in the F-GHyd2 sample, and the peak at 2*θ* = 7.6° is lessened, suggesting a decrease in the triple helices conformation in this sample. The peak at 2*θ* = 19.12° is related to the polypeptide chains' distance and suggests the amorphous structure of gelatin. In contrast, the decrease in this peak shows interactions between the SPM-β-CD polyamine and gelatin, which causes lower crystallinity. However, by inserting a higher percentage of SPM-β-CD polyamine in the gelatin hydrogel, the intensity was slightly reduced compared to pure gelatin hydrogel (Curve d). This shows that the higher percentage of SPM-β-CD in the gelatin structure increased the helical conformations and the triple helix. Considering the fact that the driving force of the alpha helix structure formation is the hydrogen bond, the aggregation of the SPM-β-CD polyamine results in the concentration of the hydrophilic groups, water adsorption, and the formation of bridges in the neighboring chains. In addition, in the F-GHyd3 case, very weak peaks at 2*θ* = 11.9°, 15.24°and 17.62° were observed related to the SPM-β-CD in the gelatin hydrogel because the SPM-β-CD content in F-GHyd3 was higher than in the F-GHyd2 hydrogel sample and the related peaks overlapped with the amorphous peak of the gelatin.^41,42^ SEM images also confirmed the XRD results. The SEM images of the all-synthesized hydrogels exhibited a reticulated porous network consisting of pores with various dimensions. Hence, the chemical cross-linking of the gelatin using EDC/NHS was performed well, and the covalent attachment of the SPM-β-CD polyamine nanoaggregates did not prevent the establishment of a porous hydrogel network. Thus, the porous cross-linked network helped the drug loading, and the subsequent release of the entrapped drug occurred satisfactorily. As shown in Fig. 4A, the presence of 10% of SPM-β-CD in the gelatin hydrogel (F-GHyd2) seems to enlarge the pores compared to the 20% of SPM-β-CD polyamine (F-GHyd3). Fig. 4A(b) displays the morphology of the F-GHyd2; a porous surface of nonuniform channels is identified.

A close exploration of the hydrogel structure (100× magnification) reveals that this polymeric network comprises small solid aggregates (35–57 nm). In some parts, the accumulations of aggregates have increased. The formation of these accumulations is likely related to the aggregations of SPM-β-CD produced by the covalent binding to the gelatin hydrogel. An increase in the SPM-β-CD percentages does not have a drastic impact on the hydrogel morphology, compared to the pure GHyd, except that, with the increase in SPM-β-CD accumulations, the walls of the hydrogel network become thicker (Fig. 4A(c)) in a way that increases the cross-linking between the gelatin chains.

The zeta potential of pure GHyd, F-GHyd2, and F-GHyd3 formulas were observed to be −8.12 (±0.52), −6.02 (±0.62) and −5.05 (±0.67) mV, respectively (Fig. S3). Zeta potentials indicate the surface charge values of the hydrogels at pH = 7.40. Such changes could be attributed to the decrease is attributed to decreasing the anionic hydroxyl and carboxylate groups on β-CD and gelatin hydrogel after the covalent attachment with SPM, creating positive amino groups.^43^

### 4.2. Choosing the best hydrogel formulation

#### 4.2.1. Hydrogel encapsulation efficiency for donepezil

The optimal percentage of hydrogel formulations was chosen based on the encapsulation efficiency measurement. In Fig. 4B, different formulations (F-GHyd1-4) with various concentrations of SPM-β-CD are compared based on the encapsulation efficiency percentage (%EE). As can be seen, the %EE in each formulation, at concentrations above 2.5 mg mL^−1^, is almost constant in a particular range; therefore, the 2.5 mg mL^−1^ concentration was chosen. On the other hand, the F-GHyd2 formulation was in a better condition than the other ones. Then, the encapsulation efficiency of various ratios of F-GHyd2 to DNP was measured to estimate the optimal DNP ratio. As seen in Table S1, by increasing the drug ratio, the encapsulation efficiency increased, and over the ratio of 25, the encapsulation efficiency had low numerical fluctuations within a specific range. Therefore, the F-GHyd2 to DNP = 25.0 was selected for further tests.

### 4.3. *In vitro* drug release evaluations

**Fig. 5:**
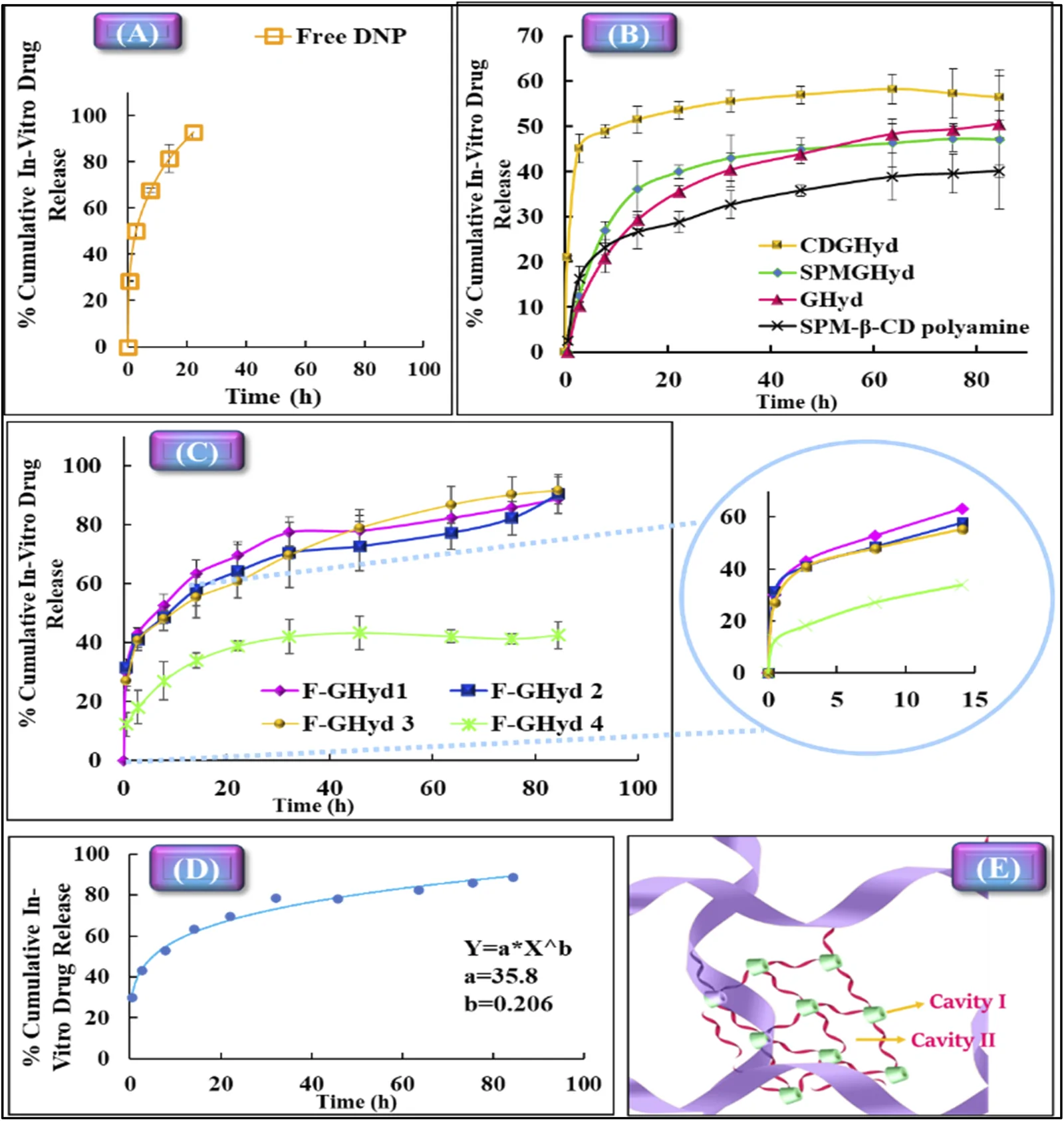
*In vitro* curve of cumulative drug release from (A) free DNP, (B) GHyd, CDGHyd, SPMGHyd and SPM-β-CD polyamine, (C) F-GHyd1, F-GHyd2, F-GHyd3 and F-GHyd4, [right: the release profile in the time range of 0–14 h], (D) mathematical fit of DNP release from F-GHyd2 *versus* time (mean ± SD, *n* = 3), (E) a schematic to represent the structural analysis of the prepared carrier and show the large and small pores.

Fig. 5 exhibits the *in vitro* DNP release profile for free DNP and from the different formulations over particular time intervals. The free DNP was found to be released completely within 22 h (Fig. 5A), whilst the *in vitro* drug release was sustained up to 84.5 h from prepared hydrogels (Fig. 5B and C). In the prepared hydrogels the nano-aggregates containing cyclodextrin that had grown on the cross-linked network of hydrogel caused the entrapment of the DNP in the carrier. Then, gelatin hydrogel could help to sustain release of the DNP.^2,44^

A cumulative percentage release of 40.14 ± 3.25% and 50.49 ± 5.91% was observed for formulations of SPM-β-CD polyamine and gelatin hydrogel alone (GHyd), respectively (Fig. 5B).

Compared with GHyd, GHyd/β-CD formulation exhibited enhanced burst and cumulative percentage DNP release (56.36 ± 4.83%), likely arising from β-CD-mediated host–guest interactions that improved DNP loading, molecular dispersion, and hydrogel hydration. Despite the elevated initial release, GHyd/β-CD retained sustained-release characteristics compared with free DNP, highlighting the contribution of β-CD inclusion cavities in regulating DNP diffusion and retention.

In contrast, GHyd/SPM displayed release kinetics comparable to those of GHyd, indicating that spermine alone exert a limited effect on DNP complexation. Rather, spermine primarily functions as a nanoarchotectural modulator through intermolecular electrostatic and hydrogen-bonding interactions. Notably, integration of spermine within the β-CD-(SPM-β-CD) functionalized hydrogel network (F-GHyd) markedly supressed burst release and generated a diffusion-regulated sustained-release profile, consistent with the formation of a more compact and interconnected nanoaggregate framework.^38,39^

As seen in Fig. 5C, the hydrogel formulations containing 8.77, 28.00, and 49.00% of SPM-β-CD polyamine (F-GHyd1, 2, and 3) show the highest release of DNP. Increasing the SPM-β-CD content (65.00% in F-GHyd4) first creates a high cross-linking density and then decreases the drug dissolution.

The burst release of DNP in the initial time intervals is observed for all formulations, followed by well-defined kinetics to obtain an efficient drug concentration level. The entrapment of the DNP molecules on the surface layer of the hydrogel network causes fast dissolution when exposed to the release environment. Due to the absence of SPM-β-CD in the GHyd formulation, it was anticipated that the initial burst release of GHyd would be more than other hydrogels. Nevertheless, the obtained release profile differed from what was expected. Probably, the absence of SPM-β-CD polyamine and the reduction of cross-linking resulted in the weak entrapment of the drug, and it was released out of the hydrogel network during the purification stage. It was observed that the initial burst release of 43.03 ± 4.60, 41.03 ± 3.58, 40.76 ± 2.44, and 18.06 ± 5.63% for F-GHyd1, 2, 3 and 4 hydrogel formulations occurred in 2.75 h, respectively while around 60% of the drug was released in 14 h (Fig. 5C, Right). The hydrogels revealed the sustained drug release with maximum release of 85.76 ± 5.01, 82.14 ± 5.60, 90.00 ± 6.18, and 40.23 ± 1.70% for F-GHyd1 to 4 hydrogel formulations in 75 h. It is possible that the inclusion complex formation of DNP with SPM-β-CD polyamine delayed the release of DNP from the hydrogels.^6,44^ The release kinetics of DNP from the hydrogels were checked first using nonlinear curve fitting to determine the line equation. As seen (Fig. 5C), the best-fitted line has an equation of *y* = *axb*. The assessment of the *in vitro* drug release is usually done by employing different mathematical equations, including Korsmeyer–Peppas, Higuchi, zero order, and first order. Applying these various kinetic models and obtaining the coefficient of regression (*R*^2^ value) (Table 1), the equation of the obtained line (*t* = 0–14 h) for the release profile indicated that the drug release followed the Higuchi model kinetics (the drug release plot *versus* square root of the time); therefore, the diffusion-controlled pattern was considered for drug release. However, a better fitting was obtained with the Korsmeyer–Peppas model (*M*_*t*_/*M*_∞_ = *kt*^*n*^).

**Table 1:** Estimation of drug release kinetic parameters and various mathematical models

Mathematical modelling	F-GHyd 1	F-GHyd 2	F-GHyd 3	F-GHyd 4	
Zero order (*Q*_*t*_ = *Q*_0_ + *K*_0_*t*)	*K*_0_ = 1.71	*K*_0_ = 1.43	*K*_0_ = 1.01	*K*_0_ = 1.56	
	*R* ^2^ = 0.91	*R* ^2^ = 0.94	*R* ^2^ = 0.92	*R* ^2^ = 0.97	
First order (log *Q* = log *Q*_0_ − *Kt*/2.303)	*K*_1_ = 0.11	*K*_1_ = 0.09	*K*_1_ = 0.04	*K*_1_ = 0.16	
	*R* ^2^ = 0.87	*R* ^2^ = 0.92	*R* ^2^ = 0.96	*R* ^2^ = 0.91	
Higuchi (*Q* = *K*_h_ × *t*^1/2^)	*K*_H_ = 0.12	*K*_H_ = 0.09	*K*_H_ = 0.10	*K*_H_ = 0.16	
	*R* ^2^ = 0.99	*R* ^2^ = 0.99	*R* ^2^ = 0.98	*R* ^2^ = 0.99	
Korsmeyer–Peppas (*M*_*t*_/*M* = *Kt*^*n*^)	*K*_K.P_ = 0.39	*K*_K.P_ = 0.38	*K*_K.P_ = 0.36	*K*_K.P_ = 0.33	
	*N* = 0.21	*N* = 0.18	*N* = 0.23	*N* = 0.27	
	*R* ^2^ = 0.99	*R* ^2^ = 0.99	*R* ^2^ = 0.99	*R* ^2^ = 0.99	
Hixson–Crowell (*W*_0_^1/3^ − *W*_*t*_^1/3^ = *kt*)	*K*_H.C_ = −0.05	*K*_H.C_ = –0.04	*K*_H.C_ = −0.02	*K*_H.C_ = −0.03	
	*R* ^2^ = 0.96	*R* ^2^ = 0.97	*R* ^2^ = 0.99	*R* ^2^ = 0.98	

In this model, *n* is the diffusional exponent, which reveals the drug release mechanism, and the formulations containing polymers could be classified based on this parameter. For hydrogels, such as those in the current research, when *n* is less than 0.45 (*n* ≤ 0.45), the Fickian-diffusion mechanism is estimated for drug release. The values 0.45 < *n* < 0.89 and *n* > 0.89 are considered for a non-Fickian transport process and the class II kinetics (drug release by an erosion-controlled mechanism), respectively.^45,46^ A structural analysis could be obtained from the drug release behavior. In the hydrogel formulations, the overall release profile was identified by two processes. First, a Korsmeyer–Peppas kinetics was assumed for about 60% of the release in 14 hours, which was obtained by the Fickian diffusion of the drug on the hydrogel surface or across the hydrogel large pores that were filled with water (cavity I). Second, a steady state situation was observed for a more extended period (84.5 h) due to the release of an excess amount of drug encapsulated in the small cavities, including small SPM-β-CD polyamine cavities, the smaller pores of the crosslinked hydrogel network, and the β-CD macrocycles that formed a genuine drug inclusion complex (cavity II) (Fig. 5E, schematic). These findings are consistent with the reports presented on the release kinetics of the cross-linked hydrogels as drug carriers. Pivato *et al.* studied the drug release kinetics of the cross-linked β-cyclodextrin prepared as nanosponge-hydrogel and revealed a Korsmeyer–Peppas kinetics based on a Fickian diffusion. According to their report, the physical and chemical characteristics of the nanosystems grown on the 3D hydrogel networks and the drug–hydrogel interactions through the 3D hydrogel architecture greatly impact on the release mechanism.^47^

In another study, the pluronic-127 polymer in the presence of either carboxymethyl cellulose or chitosan was suggested by Lungare *et al.* as an intranasal drug carrier for amantadine hydrochloride. According to their findings, because of the usage of the water-swellable polymer, the prepared formulation obeyed the Fickian-type diffusion followed by the Korsmeyere–Peppas kinetics.^48^ The concentration of the optimized formulation solution (2.5 mg mL^−1^) and the formulation containing DNP were obtained based on the mentioned results, and then DNP/F-GHyd2 was applied as the best formulation.

### 4.4. Probing the DNP/hydrogel interactions

**Fig. 6:**
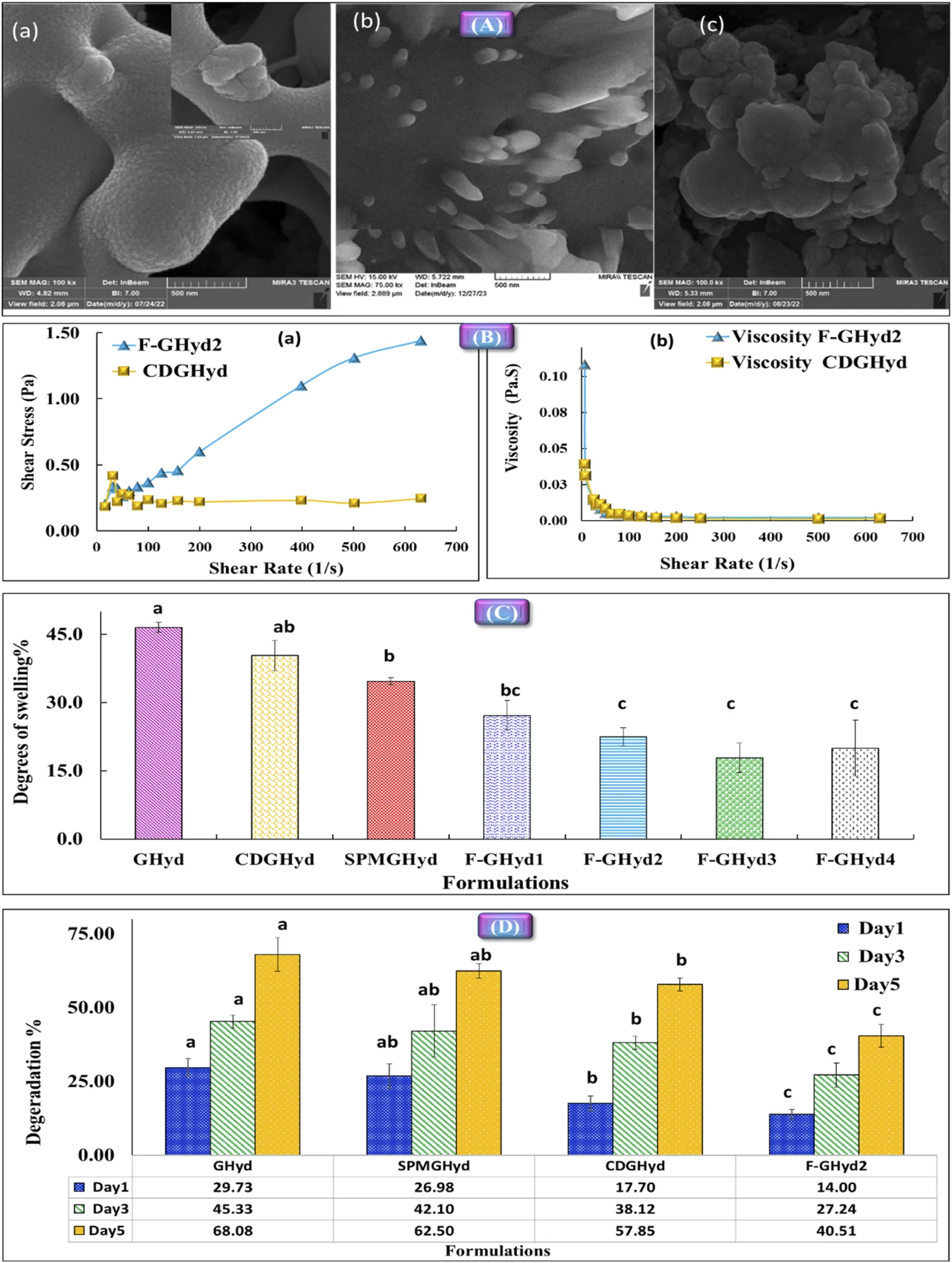
(A) SEM images of (a) dry F-GHyd2, (b) aqueous solution of F-GHyd2 without DNP and (c) aqueous solution of F-GHyd2 with DNP, (B) flow behavior of the F-GHyd2 and CDGHyd hydrogels, (a) share stress *vs.* share rate, (b) viscosity *vs.* share rate, (C) the column curve of the swelling degree of different hydrogel formulations including GHyd, CDGHyd, SPMGHyd, F-GHyd1, F-GHyd2, F-GHyd3, and F-GHyd4 and (D) *in vitro* hydrolytic degradation of GHyd, SPMGHyd, CDGHyd, and F-GHyd2 hydrogels in PBS (pH 7.4) at 37 °C for 5 days, (mean ± SD, *n* = 3). Different lowercase letters indicate statistically significant differences among formulations according to one-way ANOVA followed by Tukey's multiple comparison test (*P* < 0.05).

The FTIR spectra of free DNP, F-GHyd2 formulation, physical mixture and complexation of the DNP with F-GHyd2 hydrogel were compared to explore the DNP and hydrogel interactions precisely (Fig. S4 and S5). Considering the chemical structure of DNP (Fig. S4) and the obtained FTIR spectra, the successful incorporation of DNP into the hydrogel network was confirmed (SI). In order to evaluate the drug loading in the intended hydrogel, the SEM images of the hydrogel in the dry state, with and without a drug in the aqueous solution, were evaluated (Fig. 6A). Comparing the hydrogel in the aqueous solution (without drug) (Fig. 6A(b)) against the drug-loaded hydrogel in the aqueous solution (Fig. 6A(c)), the latter had a rougher surface than the former because the nanoparticle aggregations which had grown on the cross-linked hydrogel network (Fig. 6A(a)), were observed to be expanded (Fig. 6A(c)) in the drug-loaded hydrogel.^47,48^

### 4.5. Evaluations of the rheological features

The residence time duration in the intranasal cavity and the contact between the carrier and the olfactory epithelium or nerve are important factors affecting drug uptake. In this study, the results of rheological tests show an inverse correlation between viscosity and shear rate, revealing the shear thinning behaviour of the hydrogels (Fig. 6B).

Compared to the CDGHyd sample (the sample without SPM), the shear thinning behaviour of the hydrogel incorporating the SPM sample (F-GHyd2) was more apparent. Furthermore, in the rheograms, the relationship of the shear stress and shear rates confirmed the non-Newtonian and pseudo-plastic behaviour of the F-GHyd2 sample, while for the CDGHyd, this behaviour was not seen. Therefore, SPM-β-CD polyamine gelatin hydrogel derivatives could be considered as a favorable material for introducing new mucosal formulations.^49^

### 4.6. TGA analysis

The thermographs of hydrogels (GHyd, F-GHyd2, and F-GHyd3) are shown in Fig. S6. Considering the SPM-β-CD polyamine importance in the intended formulation design, although F-GHyd2 was chosen as the optimal formulation, the thermal degradation features related to the formulation containing higher percentages of SPM-β-CD polyamine were also investigated to support the other obtained results. As shown in Fig. S6(B and C), the F-GHyd3 thermogram differs from that of the F-GHyd2 formulation. There is a small step in the F-GHyd3 curve at 325 °C, but the maximum mass loss observed in this sample is around 297 °C, which is lower than others. This can be explained by creating new structures in the hydrogel network by increasing the percentage of SPM-β-CD polyamine, which leads to a decrease in the temperature resistance in F-GHyd3. These results were confirmed by XRD patterns.^50–52^

### 4.7. The behaviour of swelling

The results of analyzing swelling are presented in Fig. 6C, suggesting that the swelling degree of the prepared hydrogels exhibits a decreasing trend in the presence of the β-CD and the SPM-β-CD polyamine. The hydrophilic matrix of the gelatin, the functional groups of SPM and β-CD (–OH, –COOH, and –NH_2_), and the capillary forces of the hydrogel pores further facilitate water uptake by the hydrogel network. Based on the results, the higher the SPM-β-CD polyamine ratio, the higher the cross-linking density, and the lower the swelling degree. These results are consistent with the obtained results from Lu *et al.*, which described a new method to prepare chemically crosslinked gelatin-β-cyclodextrin hydrogel. They argued that the swelling degree percentage decreased by increasing the β-CD percentage.^42^ It should be noted that the sample F-GHyd4 with the higher SPM-β-CD polyamine ratio worked against the trend and exhibited a relative increase in the swelling degree compared to F-GHyd3. Based on our studies, increasing the SPM-β-CD content induced higher content of hydrophilic groups and led to the enlargement of the hydrogel network, followed by absorbing additional water molecules and increasing the swelling degree.^51,53^ The *in vitro* hydrolytic degradation behavior of the prepared hydrogels was evaluated in PBS (pH 7.4) at 37 °C over five days, and the results are presented in Fig. 6D. All formulations exhibited a gradual increase in degradation with increasing incubation time. Among the investigated hydrogels, GHyd showed the highest degradation, reaching 68% after 5 days, whereas F-GHyd2 exhibited the lowest degradation (40%), indicating improved structural stability. The intermediate degradation behavior observed for SPMGHyd (62%) and CDGHyd (58%) indicates that spermine and β-CD individually contributed to reducing hydrolytic degradation. Notably, their conjugation within the SPM-β-CD-functionalized hydrogel further suppressed degradation, suggesting the formation of a more compact and interconnected nanoaggregate network with enhanced resistance to water penetration and polymer chain erosion, consistent with previous reports on crosslinked hydrogel systems.^39^

### 4.8. Pharmacokinetic explorations

As previous studies have shown, the intranasal administration of DNP in the form of different formulations such as solid lipid nanoparticles ^6^ thiolated chitosan hydrogel ^16^ nanogels ^4^ lipid nanostructure coated with chitosan ^54^ the polymeric nasal film containing Hydroxypropylmethylcellulose (HPMC), Methyl-β-Cyclodextrin (Me-β-CD) and poly (ethylene glycol) 400 (PEG400)^55^ could improve the pharmacokinetic parameters in comparison to free DNP.

#### 4.8.1. HPLC conditions

**Fig. 7:**
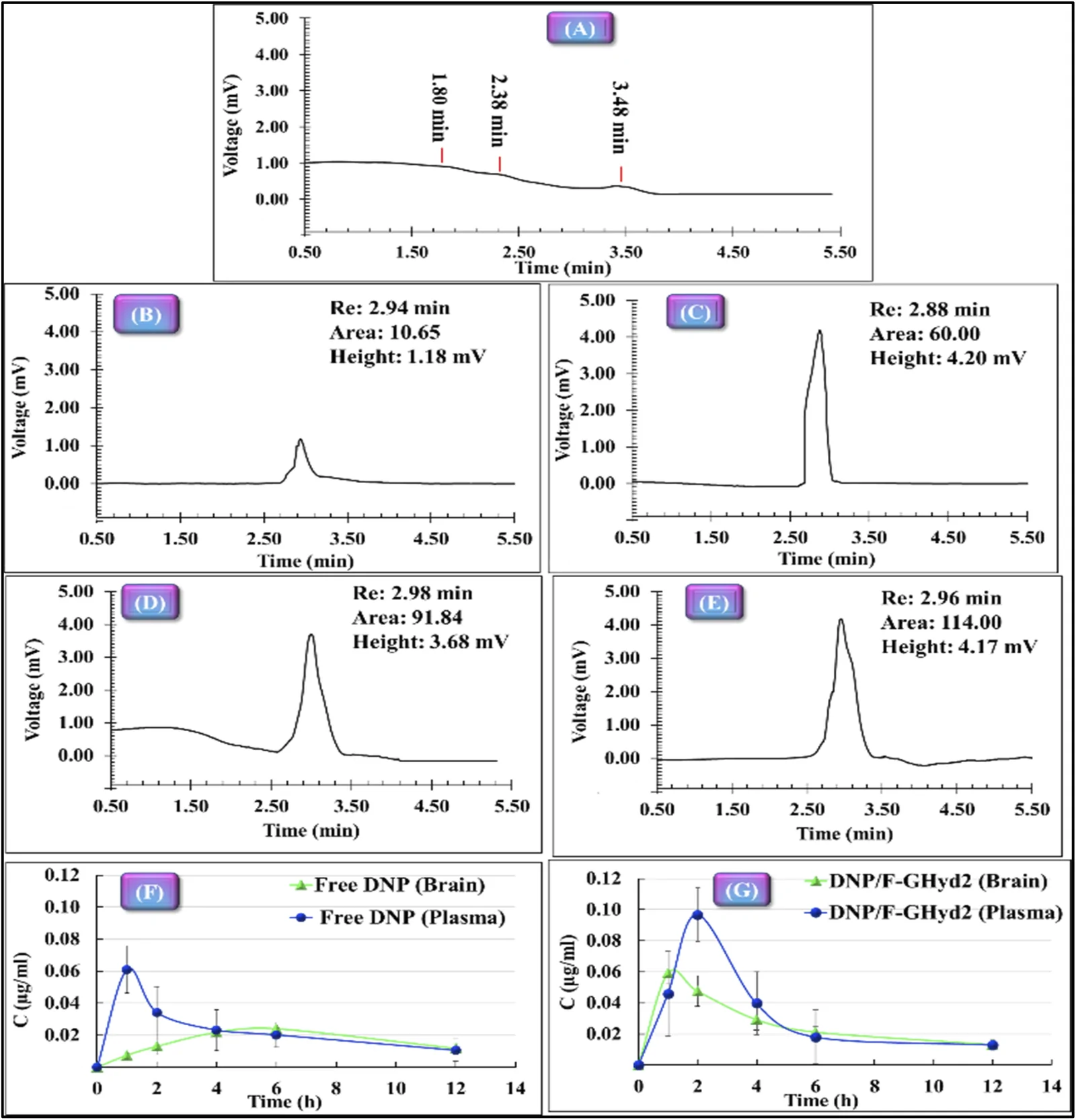
HPLC chromatograms of (A) blank matrix, (B) and (C) DNP standard solutions 4.0 and 20.0 ng mL^−1^, (D) extracted DNP content in the brain, (E) DNP extracted from the brain tissue that spiked with 20 ng mL^−1^DNP after intranasal administration of DNP/F-GHyd2, the profile of the DNP concentration against time in plasma and the brain (F) free DNP solution and (G) DNP-loaded hydrogel formulation (DNP/F-GHyd2) (mean ± SD, *n* = 3).

The presence of DNP in the rat brain was confirmed by HPLC analysis. Representative blank matrix chromatograms (Fig. 7A) showed no endogenous interfering peaks at the retention time of DNP when compared with the corresponding standard and spiked samples, thereby confirming the specificity and selectivity of the analytical method. This observation confirms the selectivity of the method. The chromatograms of DNP standard solutions (4.0 and 20.0 ng mL^−1^) are exhibited in Fig. 7B and C. The retention time of DNP was determined to be approximately 2.90 min, which is in agreement with previously reported under comparable chromatographic conditions.^5^ The chromatograms resulted from brain samples containing DNP collected 2 h after nasal administration of DNP/F-GHyd2 to the rats are shown in Fig. 7D and E. The peaks corresponding to DNP were distinct, with the retention times of approximately 2.98 min. The brain sample (Fig. 7D) in comparison to the spiked brain samples (Fig. 7E) did not show interfering peaks at the retention times corresponding to DNP (*R*_e_ = 2.96 min), indicating the specificity of the assay.

Therefore, the two peaks resulting from the brain and spiked samples were compatible with each other and confirmed the presence of DNP in the brain.

To estimate the linearity of the technique, calibration curves were established using plasma samples containing 1.0–100.0 ng mL^−1^of DNP and the linear relationship *y* = 2.46*x* + 0.03 was generated between the peak area of DNP and the corresponding DNP concentrations (*R*^2^ = 0.9971). The high value of the correlation coefficient (*R*^2^) confirmed the validation of the calibration curve linearity. The precision values (%RSD) for intra-day measures were < 7.10%. The concentration profiles of DNP in plasma and brain (mean ± SD) in the rats after the intranasal administration of free and loaded DNP are shown in Fig. 7C and D. Different pharmacokinetic parameters were calculated for free and loaded DNP formulations (DNP/F-GHyd2) can be seen in Table 2. After administrating the DNP/F-GHyd2 formulation intranasally, comparing the tmax in the brain and plasma shows that the tmax for the brain (1 h) was lower than that for plasma (*t*_max_ = 2 h), which could indicate the superiority of the F-GHyd2 formulation for DNP intranasal administration.^6^

**Table 2:** Pharmacokinetic parameters of plasma and brain upon intranasal administration of the free DNP solution and DNP/F-GHyd2 (mean ± SD, *n* = 3). Statistical significance between the free DNP solution and DNP/F-GHyd2 in brain tissue was determined using an unpaired two-tailed Student's *t*-test. **P < 0.01; ***P < 0.001

Pharmacokinetic parameters as mean ± SD (*n* = 3)	Free DNP		DNP/F-GHyd2	
	Brain	Plasma	Brain	Plasma
*t*_Max_ (h)	6	1	1	2
*C*_Max_ (µg mL^−1^)	0.024 (±0.005)	0.074 (±0.007)	0.059 (±0.006)**	0.097 (±0.017)
AUC (µg h mL^−1^)	0.20 (±0.05)	0.28 (±0.02)	0.31 (±0.01)***	0.45 (±0.14)
AUMC (µg h^2^ mL^−1^)	1.24 (±0.44)	1.23 (±0.06)	1.6 (±0.23)	1.84 (±0.68)
MRT (h)	5.89 (±0.81)	4.46 (±0.63)	4.82 (±0.49)	4.03 (±0.46)
*K*_e_ (h^−1^)	0.31 (±0.07)	0.23 (±0.04)	0.18 (±0.02)	0.19 (±0.03)
*t*_1/2_ (h)	2.36 (±0.52)	3.04 (±0.51)	5.33 (±0.09)	3.61 (±0.50)

Furthermore, when the tmax points were compared in plasma, free DNP and DNP/F-GHyd2 attained maximum values within 1 h and 2 h, respectively. Then, the tmax in the brain was found to be 6 h for free DNP and 1 h for DNP/F-GHyd2 (Fig. 7B and C). The *C*_max_ values in the brain (0.059 (±0.006) µg mL^−1^) and plasma (0.097 (±0.017) µg mL^−1^) for DNP/F-GHyd2 were observed to be remarkably higher than those for free DNP in the brain (0.024 (±0.005) µg mL^−1^) and plasma (0.074 (±0.007) µg mL^−1^)). It shows the effectiveness of the F-GHyd2 formulation in increasing the DNP concentration in the brain to 59.13% compared to free DNP through the nose-to-brain route. The free DNP showed lower tmax than the DNP/F-GHyd2 in plasma. In fact, free DNP attained systemic circulation faster than the loaded DNP *via* the internasal route. The DNP/F-GHyd2 formulation containing absorption enhancers (gelatin hydrogel and SPM-β-CD) caused accumulation of the drug in the nasal mucosa, released the drug slowly into the circulation, and delayed the *t*_max_. On the contrary, it increased the *C*_max_ compared to the free DNP. Moreover, the DNP/F-GHyd2 formulation had higher AUC0-12 h values than the free DNP solution.^56^ The AUC0-12 h of DNP in the brain for the DNP/F-GHyd2 was 35% greater than for free DNP. The obtained results revealed that the penetration of the drug into the brain from the nasal mucosa takes place by two different pathways: systemic and olfactory pathways. In the systemic pathway, the drug goes into systemic distribution after intranasal administration, permeating the blood–brain barrier (BBB) and reaching the brain. In the second pathway, the drug passes through the olfactory mucosa from the intranasal cavity to the cerebrospinal fluid (CSF) or the brain. The results showed better bioavailability of DNP administrated by DNP/F-GHyd2 formulation in the brain compared to the free DNP solution.^57^ The remarkably enhanced bioavailability of the DNP in the F-GHyd2 formulation in compared to the free drug solution may be due to the improved drug permeability through the nasal mucosa by gelatin and spermine-β-CD. Due to the higher aqueous solubility and acceptable safety of the functionalized β-CDs and polymers based on β-CDs such as hydroxypropyl-β-cyclodextrin, sulfobutyl-ether-β-cyclodextrin, dimethyl-β-cyclodextrin and β-Cyclodextrin-poly (β-Amino Ester), they have been extensively used to improve absorption in nasal formulations.^58–60^ The various reasons for enhancing the intranasal delivery of the DNP/F-GHyd2 formulation to the brain can be found in previous reports. The aminated gelatin microspheres prepared by Wang *et al.* exhibited remarkable enhancement in the nasal absorption of insulin. According to their reports, the absorption of water from the mucous membrane in the nasal cavity by the hydrogel causes dehydration in the membrane cells, affects the junctions, opens the nasal epithelium cells, and increases permeability.^61^ As reported by Seki *et al.*, the presence of SPM and the introduction of the amine groups in the hydrogel formulation cause electrostatic interactions between the hydrogel and the epithelial layer of nasal mucus. Then, the interactions increase the permeability of the tight junctions for the drug.^62^

Moreover, the desired effect of β-CD in the hydrogel formulation has been studied in previous literature as an absorption enhancer for intranasal delivery of drugs. Cyclodextrins can enhance the drug solubility and facilitate the drug permeability through the epithelia cells. Because of the ability to form inclusion complexes with the cholesterol molecules, CDs could reversibly eliminate the cholesterol from the membrane's outer bilayer and then cause the tight junctions to open and enhance the drug permeability for a short time.^58^

### 4.9. Toxicity evaluations

Considering the importance of drug carriers' safety for clinical usage, it is crucial to check the carriers' toxicity. In this regard, brain tissue histopathological assessments were carried out to explore this point. The histopathological images did not show any microscopic changes or pathological lesions in the brain tissue of the animals treated with DNP/F-GHyd2 suspension compared to the animals treated with the free DNP solution (Fig. S7).

### 4.10. Stability evaluations

#### 4.10.1. DLS analysis

The stability studies were done for the DNP/F-GHyd2 formulation. The particle size results are shown in Table S2 (Fig. S8). It can be seen in Table S2 that the addition of DNP to the aqueous colloidal of F-GHyd2 caused an increase in the average hydrogel particle size, from 175 (with 69% of the particles having a size less than 246 nm) to 217 nm, (with 69% of the particles having a size less than 372 nm). It is probably the result of the DNP inclusion complex formation onto the hydrogel surface. After storing the hydrogel colloidal formulation (DNP/F-GHyd2), after two weeks, one month, and three months, its sizes were obtained to be about 261 (58% (<499 nm)), 422 (22% (<422 nm), and 84% (<1113 nm)) and 1115 nm (67% (<1178 nm), respectively (Table S2). Although their sizes increased over time, they still retained the necessary standards to deliver drugs from the nose to the brain so that the size was not so small as to pass through the tracheobronchial with the airflow and not so big as to bypass directly by way of the olfactory and trigeminal nerves.^16,63^

#### 4.10.2. Radical scavenging ability

Considering the observed variations in the particle size over time, the DPPH assay was carried out to explore the efficacy of the storage drug-loaded F-GHyd2 sample. Some evidence indicates that the tau protein and β amyloid (Aβ) accumulation and oxidative stress are the factors involved in the beginning and continuation of AD. Recent researches show that oxidative stress is a major factor in the progression of AD. Overproduction of reactive oxygen species (ROS) causes oxidative stress, which, over time, results in the accumulation of neuronal damage to the brain tissue of AD patients.

ROS, such as hydroxyl radical, superoxide anion, and hydrogen peroxide, are a group of reactive molecules, ions, and free radicals obtained from O2. The brain needs a lot of energy to preserve the neurons and glial cells' function. As a result, the O2 consumption by the brain is considerably high. The production of ROS rising from the high consumption of O2 by brain tissue is one of the reasons for the decreased performance in the cognitive function seen in AD. Excessive ROS would produce functional changes in synapses. Considering the influential role of oxidative stress in the progression of AD, the utilization of antioxidants along with the drugs can significantly limit the progression of AD.^64,65^

The potential anti-inflammatory effects and free radical scavenging of spermine in mitochondria, the nucleus, and the brain have been reported in the literature. Therefore, the gelatin hydrogel containing spermine/cyclodextrin is expected to be able to improve the antioxidant activity of the prepared DNP nasal delivery system by reducing ROS stress, which results in slowing brain damage progression. According to the points mentioned about the antioxidant effect of the prepared hydrogel, it can be concluded that the application of this hydrogel for DNP delivery to the brain improves the efficiency of the delivery system and that monitoring the radical scavenging ability of the designed carrier for DNP is an important parameter in order to check the carrier efficiency *in vitro*.^12,15^

At first, the radical scavenging activities of F-GHyd2 hydrogel and free DNP were studied (Fig. S9(A)). The F-GHyd2 hydrogel caused considerable scavenging activity (concentration = 2.5 mg mL^−1^, 40.29% ± 3.06), which increased remarkably (72.33% ± 3.43) in the presence of DNP (concentration = 2.5 µg mL^−1^) (DNP/F-GHyd2). For comparison, the scavenging activity of donepezil alone was also measured (concentration = 2.5 µg mL^−1^, 30.86% ± 3.91) (Fig. S9(B)). This result was consistent with the report of Munishamappa *et al.* They used a DPPH antioxidant method for different DNP concentrations and obtained the free radical inhibitory of 33.5% at 10 µg mL^−1^.^66^ The time stability evaluations were performed based on the DPPH assay for DNP/F-GHyd2. The DPPH radical scavenging was assessed in three months for DNP/F-GHyd2, where in comparison to the initial sample, the radical scavenging activity of the formulation decreased slowly. However, after three months, it still showed high antioxidant properties (68.26% ± 4.01) (Table S2). Thus, the designed formulation maintained its effectiveness, and the observed changes in particle size (based on DLS results) did not significantly affect its performance.

#### 4.10.3. *In vitro* release study

**Fig. 8:**
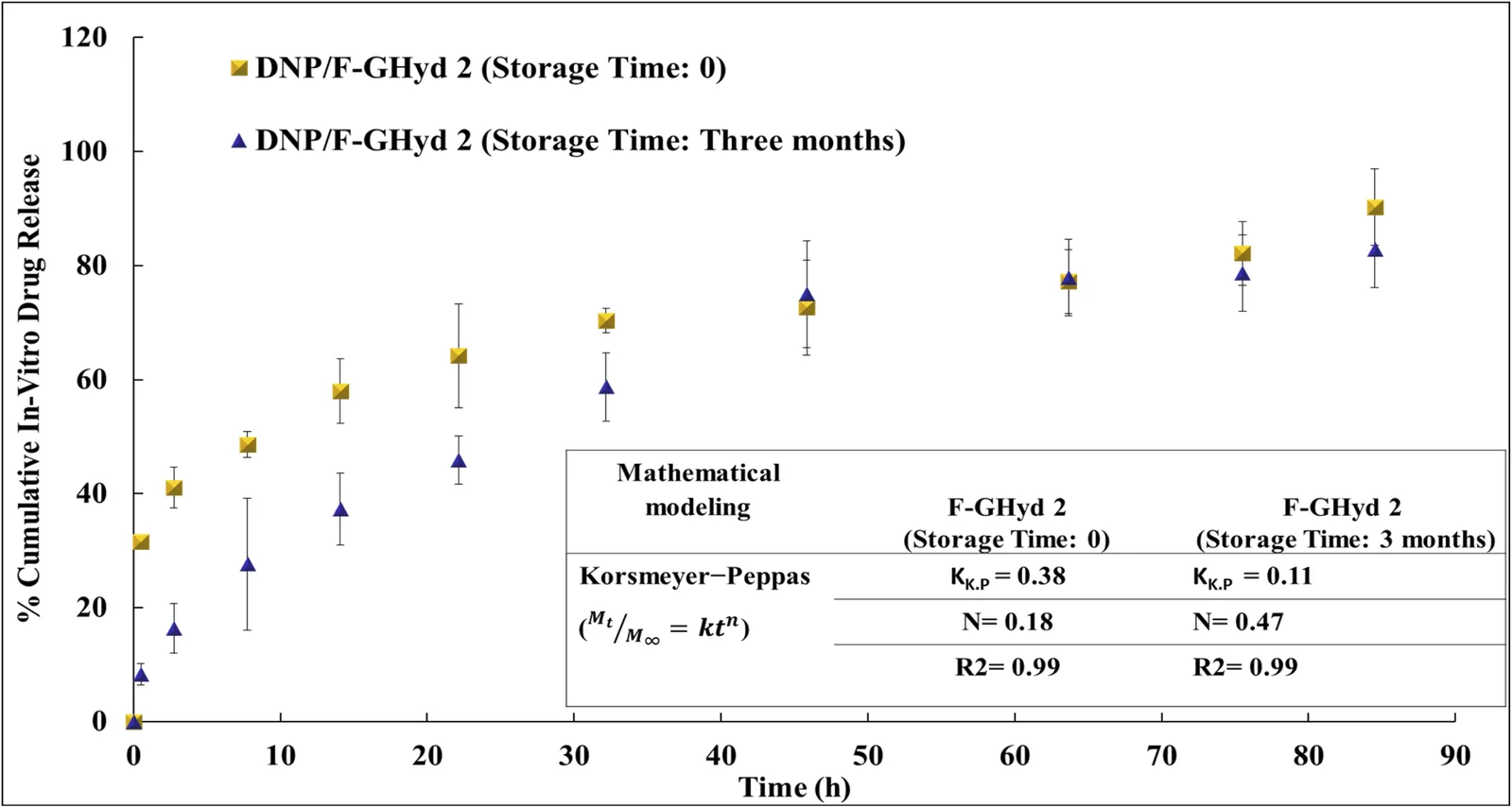
*In vitro* drug release assessment of the DNP/F-GHyd2 formulation at time storage 0 and after 3 months storage (effect of storage time on the *in vitro* drug release) [inset: comparison of the release kinetics parameters obtained by the Korsmeyer–Peppas model] (mean ± SD, *n* = 3).

In order to have more precise insight into the stability of the hydrogel formulation as a nasal carrier, the release of the drug was evaluated *in vitro* after 3-months storage. The cumulative percentage release of DNP from F-GHyd2 was obtained at 82.80 ± 8.04% (Fig. 8) after 3-months storage at 5 °C. Although there was a relative decrease compared to time = 0, this is still an acceptable value. Nevertheless, the DNP release displayed different behavior at the initial intervals in the drug release profile (0.50–32 h). As mentioned, the Korsmeyere–Peppas kinetics model with a Fickian diffusion was described for drug release from the prepared hydrogel formulations at week = 0. After 3 months, due to the increase in the *n* value (Korsmeyere–Peppas exponent) to 0.47, the release mechanism changed to anomalous mass transport (Fig. 8). The solvent diffusion rate and the polymer chains relaxation rate are the two main factors affecting the diffusional process to illustrate the anomalous release. When the diffusion rate is slower than the rate of the polymer chain relaxation, it is considered to be Fickian (Case I). For non-Fickian diffusion, there are two classifications for diffusion based on the rate of solvent diffusion: Case II transport and anomalous diffusion. The Case II transport happens when the solvent diffusion rate is much higher than the polymer chain relaxation rate. The anomalous diffusion occurs when the rate of the solvent diffusion and the polymer relaxation have relatively the same values.^47,67,68^

Therefore, polymer relaxation exhibited an essential role at the initial intervals for DNP release from storage formulation (in 3 months). In order to check out the stability, the formulation was kept in the liquid state at 5C. It is possible that, during the storage, some forces, such as repulsion between carboxylate ions (COO−), changed the interactions between the polymer chains in the hydrogel networks and between the hydrogel and the drug in a way that they altered the drug release mechanism from Fickian-diffusion to an anomalous mechanism.^69^

## 5. Discussion

Recently, polymeric and lipid-based carriers have attracted considerable attention for nose-to-brain drug delivery due to their potential to overcome the limitations imposed by the blood–brain barrier. Polymeric and lipid-based nanocarriers have been extensively investigated for nose-to-brain drug delivery because of their ability to improve drug transport across the blood–brain barrier. However, many existing systems, particularly lipid-based formulations, suffer from rapid mucociliary clearance and limited residence time within the nasal cavity, thereby reducing drug absorption efficiency.^9,27,54,55,70^ Consequently, the rational exploitation of hydrogel-based systems is increasingly regarded as an effective strategy for the development of advanced drug delivery carriers. In this context, the modified nasal hydrogel formulation (F-GHyd2) developed in the present study; comprising gelatin, β-CD and spermine; demonstrates considerable potential as an intranasal delivery platform.

Beyond its controlled biodegradability, the proposed formulation provides a structurally robust yet biocompatible supramolecular matrix for efficient nose-to-brain delivery. Importantly, F-GHyd2 combines sustained drug release with the intrinsic antioxidant and anti-inflammatory characteristics of its constituent biomaterials, which may further improve therapeutic efficacy and mucosal compatibility.

SEM images revealed that the prepared F-GHyd exhibits a highly porous and interconnected network composed of crosslinked gelatin chains decorated with spermine-β-cyclodextrin-polyamine (SPM-β-CD) nanoaggregates. This hierarchical architecture provides a large specific surface area and abundant internal porosity, enabling efficient loading of DNP both within the hydrogel matrix and inside the SPM-β-CD nanoaggregates.

*In vitro* release studies of F-GHyd2 demonstrated an initial DNP release governed by Fickian diffusion for up to 14 h, followed by a sustained release phase at longer time points. The encapsulation and retention behaviour of DNP within the hydrogel matrix are likely governed by multiple non-covalent interactions, including β-CD-mediated host–guest inclusion, hydrogen bonding, and diffusion-controlled entrapment within the interconnected hydrogel pores and SPM-β-CD nanoaggregate cavities. These proposed mechanisms are supported by the obtained release kinetics, FTIR spectral changes, and the sustained-release characteristics of the developed formulations. The TGA results suggest that incorporation of SPM-β-CD polyamine modulates the thermal degradation behavior and network organization of the hydrogel system, confirming the structural influence of the nanoaggregate component within the gelatin hydrogel matrix.

The gradual decrease in swelling degree upon incorporation of β-CD, spermine, and increasing SPM-β-CD polyamine content suggests the formation of a progressively denser nanoaggregate-mediated intermolecularly crosslinked hydrogel network stabilized through hydrogen bonding, host–guest interactions, and polyamine-mediated intermolecular associations. Interestingly, F-GHyd4 deviated from this trend despite its higher SPM-β-CD content, implying that excessive nanoaggregate incorporation may modulate network organization and alter the balance between intermolecular interactions and hydration behaviour, consistent with the corresponding release and TGA profiles.

The hydrolytic degradation study provided additional insight into the structure–property relationship governing the performance of the developed hydrogel system. As expected, GHyd exhibited the highest degradation owing to its relatively loose network architecture and higher swelling capacity, which facilitated water penetration and accelerated polymer chain erosion. The incorporation of spermine or β-CD individually resulted in a moderate reduction in degradation, indicating that each component contributed to network stabilization through additional intermolecular interactions and increased structural cohesion. More importantly, the SPM-β-CD-functionalized hydrogel (F-GHyd2) exhibited the lowest degradation rate, demonstrating that the conjugation of spermine with CM-β-CD generated a more compact and interconnected nanoaggregate architecture that effectively restricted water diffusion into the polymer matrix. This behavior is in excellent agreement with the swelling results, where F-GHyd2 showed a lower equilibrium swelling ratio, confirming the formation of a denser crosslinked network. Likewise, the enhanced thermal stability observed by TGA further supports the increased structural integrity of the SPM-β-CD-functionalized hydrogel. Such a stabilized nanoaggregate network also explains the markedly suppressed burst release and prolonged diffusion-controlled release profile observed in the release experiments, where the interconnected polymeric framework and β-CD inclusion cavities cooperatively regulated drug diffusion. Collectively, these findings demonstrate that the improved performance of the proposed carrier is not merely a consequence of introducing spermine or β-CD as individual components, but rather originates from the supramolecular nanoaggregate architecture generated by their chemical conjugation. Importantly, the present hydrogel system is distinguished from previously reported intranasal donepezil carriers by its rational molecular design, in which chemically conjugated spermine-β-cyclodextrin (SPM-β-CD) nanoaggregates are integrated within a gelatin hydrogel network to create a unique hierarchical supramolecular architecture. To the best of our knowledge, this is the first report describing a chemically engineered gelatin hydrogel incorporating spermine-β-cyclodextrin nanoaggregates as a biodegradable supramolecular platform for sustained intranasal delivery of donepezil. Rather than functioning merely as structural additives, the SPM-β-CD nanoaggregates act as molecular regulators of the hydrogel architecture, simultaneously governing swelling behavior, hydrolytic degradation, thermal stability, drug-release kinetics, and ultimately the *in vivo* nose-to-brain delivery performance. These findings demonstrate that the molecular organization of the SPM-β-CD nanoaggregates within the gelatin network, rather than the individual building blocks themselves, governs the physicochemical properties, biodegradation behavior, and functional performance of the developed carrier, thereby establishing a rational molecular design strategy for next-generation biodegradable intranasal hydrogel systems. From a pharmaceutical perspective, this controlled biodegradation behavior is particularly advantageous for intranasal drug delivery, as it enables the hydrogel to preserve its structural integrity throughout the sustained drug-release period while undergoing gradual biodegradation thereafter, thereby minimizing long-term tissue accumulation following repeated administration.

The synergistic integration of β-CD and spermine within the engineered hydrogel network facilitates host–guest interactions while simultaneously improving the structural organization of the carrier, thereby contributing to sustained drug release and enhanced nasal absorption. Given that neurodegenerative disorders such as Alzheimer's disease are strongly associated with oxidative stress, the observed free radical scavenging activity of DNP/F-GHyd2 (72.33 ± 3.43%) indicates the intrinsic antioxidant capacity of the developed hydrogel system. Although the present study did not directly evaluate oxidative stress *in vivo*, this antioxidant property may provide additional therapeutic potential for mitigating oxidative stress associated with Alzheimer's disease. In addition to these physicochemical characteristics, the enhanced brain accumulation observed following intranasal administration also suggests that the molecular design of the hydrogel may facilitate drug transport to the central nervous system. Although the precise transport mechanism was not investigated in the present study, previous reports suggest that polyamine-functionalized nanocarriers may promote cellular uptake, membrane permeation, and transcytosis across biological barriers, which may partly explain the improved brain delivery observed for F-GHyd2. Nevertheless, dedicated biological investigations, including epithelial transport, mucoadhesion, and barrier integrity studies, will be required to verify these proposed mechanisms in future work.

*In vivo* studies further confirmed the therapeutic potential of the developed hydrogel system. Intranasal administration of DNP/F-GHyd2 resulted in significantly higher DNP accumulation in the brain compared to free DNP solution, highlighting the efficiency of the proposed nose-to-brain delivery strategy. Histopathological evaluation demonstrated that DNP/F-GHyd2 containing 0.5 mgmL^−1^ DNP was well tolerated, with no observable tissue damage, confirming its safety profile. Notably, intranasal administration enables lower drug doses to achieve therapeutic efficacy compared to systemic routes, owing to direct drug localization within the central nervous system.^71–73^ Recent advances in intranasal delivery have increasingly focused on the use of biopolymers to overcome mucosal barriers. For instance, Jadhav *et al.* reported methyl-β-cyclodextrin (M-β-CD) microparticles that enhanced nasal permeability by disrupting the mucus barrier, thereby improving the delivery of anti-tuberculosis drugs.^74^ Similarly, Ruan *et al.*, developed a microneedle-based nasal patch using tannic acid cross-linked gelatin and hyaluronic acid microneedle matrix loaded with cyclodextrin/MOF-loaded huperzine A, successfully addressing rapid mucociliary clearance through prolonged mucosal contact.^75^ Despite these advances, while nose-to-brain delivery remains a highly promising strategy to circumvent first-pass metabolism and reticuloendothelial system uptake, the clinical translation of intranasal therapeutics formulations still faces significant challenges and requires further optimization.^76–78^

## 6. Conclusion

The present study reports, to the best of our knowledge, the first chemically engineered gelatin hydrogel incorporating spermine-β-cyclodextrin (SPM-β-CD) nanoaggregates as a biodegradable supramolecular platform for intranasal delivery of donepezil hydrochloride. Formation of the hierarchical SPM-β-CD nanoaggregate architecture established a clear structure–property–function relationship in which the molecular organization of the hydrogel network governed swelling behavior, hydrolytic biodegradation, thermal stability, sustained drug release, and brain-targeting performance. The optimized formulation (DNP/F-GHyd2) exhibited sustained diffusion-controlled release together with significantly enhanced brain accumulation of donepezil following intranasal administration, confirming the effectiveness of the proposed molecular design for nose-to-brain delivery. Moreover, the hydrogel demonstrated excellent *in vivo* biocompatibility without detectable histopathological alterations and simultaneously exhibited antioxidant activity, offering additional therapeutic potential for mitigating oxidative stress associated with Alzheimer's disease. Importantly, the significance of this work extends beyond the development of another intranasal formulation. Rather, it demonstrates that rational molecular engineering of SPM-β-CD nanoaggregates within a gelatin network can be exploited to regulate the physicochemical properties and pharmaceutical function of biodegradable hydrogels through a unified supramolecular architecture. This molecular design principle may provide a generalizable platform for future intranasal delivery of other central nervous system therapeutics.

Future investigations should focus on evaluating long-term therapeutic efficacy in relevant Alzheimer's disease models together with comprehensive pharmacokinetic studies, including regional brain distribution within different brain regions (*e.g.*, olfactory bulb, cortex, and hippocampus), optimization of formulation-dependent parameters (nasal deposition, sprayability, and aerosol performance), and clinical translation of this supramolecular hydrogel platform.

## Ethical statement

All animal procedures were performed in accordance with the Guidelines for Care and Use of Laboratory Animals of Shiraz University of Medical Sciences (Iran). Animals were handled according to the animal handling protocol approved by institutional ethics committee (code: 99027912). The protocols using animals in the current study were also conducted according to the ARRIVE guidelines (reference: https://doi.org/10.1371/journal.pbio.3000410).

## Author contributions

Sedigheh Hashemnia: supervision, project administration conceptualization, methodology, validation, formal analysis, investigation, writing – review and editing. Zaynab Mokhtari: conceptualization, methodology, validation, formal analysis, investigation, writing – original draft. Reza Heidari: validation, formal analysis, writing – review and editing. Mohammad Hossein Mokhtari: investigation, formal analysis, conceptualization.

## Conflicts of interest

There are no conflicts to declare.

## Supplementary Material

RA-OLF-D6RA01040G-s001

## Data Availability

The data that support the findings of this study are available from the corresponding author upon reasonable request. Supplementary information (SI): the characterization data of carboxymethyl-β-cyclodextrin (CM-β-CD) by FT-IR and 1H NMR spectroscopy Fig. S1); UV-vis calibration spectra of donepezil (DNP) (Fig. S2); zeta-potential measurements (Fig. S3); encapsulation efficiency data (Table S1); chemical structure of donepezil (DNP) (Fig. S4) FT-IR analysis of DNP/hydrogel interactions (Fig. S5); thermogravimetric analysis (TGA/DTG) (Fig. S6); histopathological images of rat brain tissue (Fig. S7); time-dependent DLS/stability data (Fig. S8, Table S2); DPPH radical-scavenging figs (Fig. S9). See DOI: https://doi.org/10.1039/d6ra01040g.
