# Supplementary material for: Design of a spermine-β-cyclodextrin functionalized gelatin nanoaggregate hydrogel for nose-to-brain delivery of donepezil
Source: RSC Adv. 2026 Sep 1;16(42):51387–410. doi: 10.1039/d6ra01040g (PMC13531293; doi:10.1039/d6ra01040g)
Supplement: Supplementary file 1 [file RA-016-D6RA01040G-s001.pdf]

Supplementary Information (SI) for RSC Advances.
This journal is © The Royal Society of Chemistry 2026

Supporting Information

Design of a Spermine-β-Cyclodextrin Functionalized Gelatin Nanoaggregate Hydrogel for Nose-to-Brain Delivery of Donepezil

Zaynab Mokhtari[a,b], Sedigheh Hashemnia*[a], Reza Heidari[c], Mohammad Hossein Mokhtari[d]

[a] Department of Chemistry, Faculty of Nano and Bio Science and Technology, Persian Gulf University, Bushehr 75169, Iran.

[b] Department of Chemical Industry, Technical and Vocational University (TVU), Bushehr, Iran

[c] Pharmaceutical Sciences Research Center, Shiraz University of Medical Sciences, Shiraz, Iran

[d] Department of medicinal chemistry, school of pharmacy, Mashhad University of Medical Sciences, Iran

* Corresponding author's E-mail addresses: shashemnia@pgu.ac.ir (S. Hashemnia)

Characterization of the synthesized carboxylmethyl β- cyclodextrin (CM- β-CD)

Fig. S1 (A) shows the FT-IR spectrum of CM- β-CD. The peaks around 2925,1149, 1076, 1014, and 935 cm^{-1} are associated with the main β-CD skeletal structure. These are assigned to the aliphatic C-H stretches, C–C/C–O and C–O–C stretching vibrations, and 1, 4-glycosidic structural skeleton of β-CD. The peak in the region of 1791 cm^{-1}, and the peaks in the regions of 1567 and 1410 cm^{-1} are connected to the stretching vibration of carbonyl groups and the symmetric and asymmetric stretch of carboxyl groups, respectively, which confirms the carboxymethylation of β –CD.

Fig. S1 (B), shows H NMR spectrum of CM- β-CD. The peak at 4.96 ppm corresponds to the CH anomeric proton (H-1), while a series of peaks between 3.46 and 4.50 ppm are assigned to the CH protons of H-4, H-3, H-6, H-2, H-7 (-CO-CH_2, methylene protons of carboxymethyl group), and H-5. The H NMR experiment was performed on a 300 MHz spectrometer (Bruker, Germany) in D_2O solvent.

FT-IR

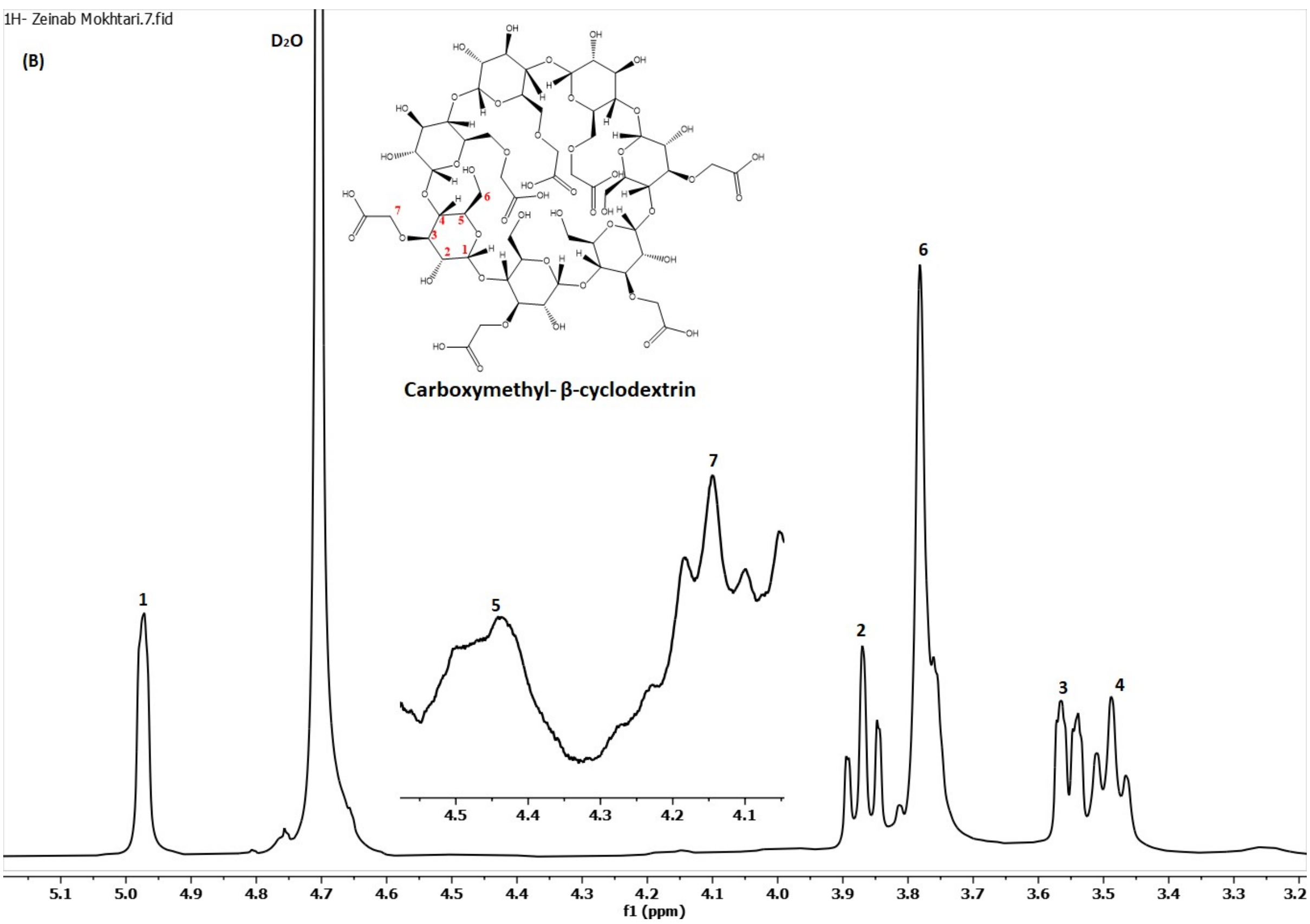

Fig. S1 (A) FT-IR spectrum of CM- β-CD. (B) H NMR of CM-β-CD.

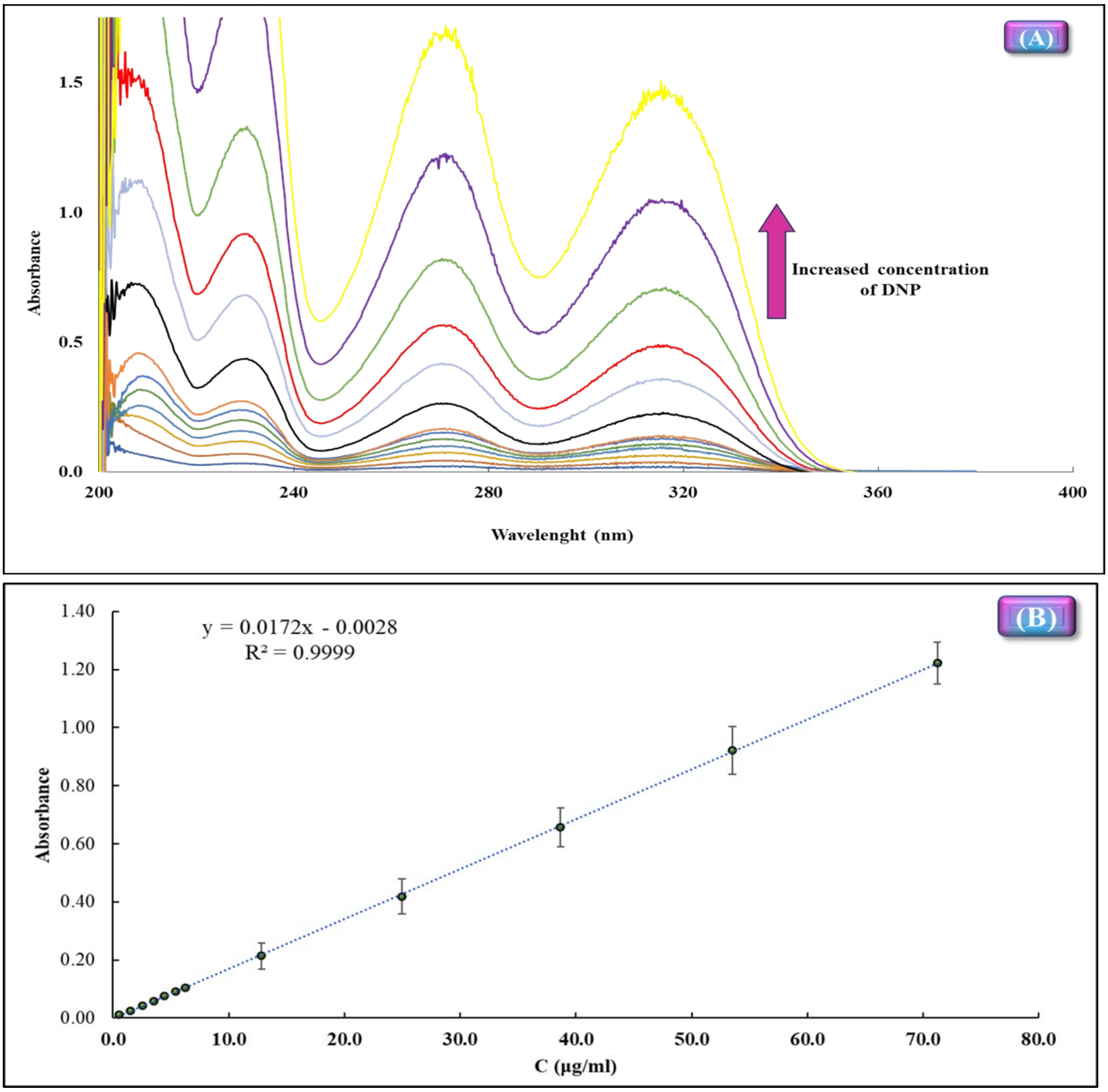

Fig. S2 UV/Visible absorbance spectra of different concentration of DNP (0.55 to 71.00 µg/mL) in PBS pH 7.40 (mean ±SD, n=3).

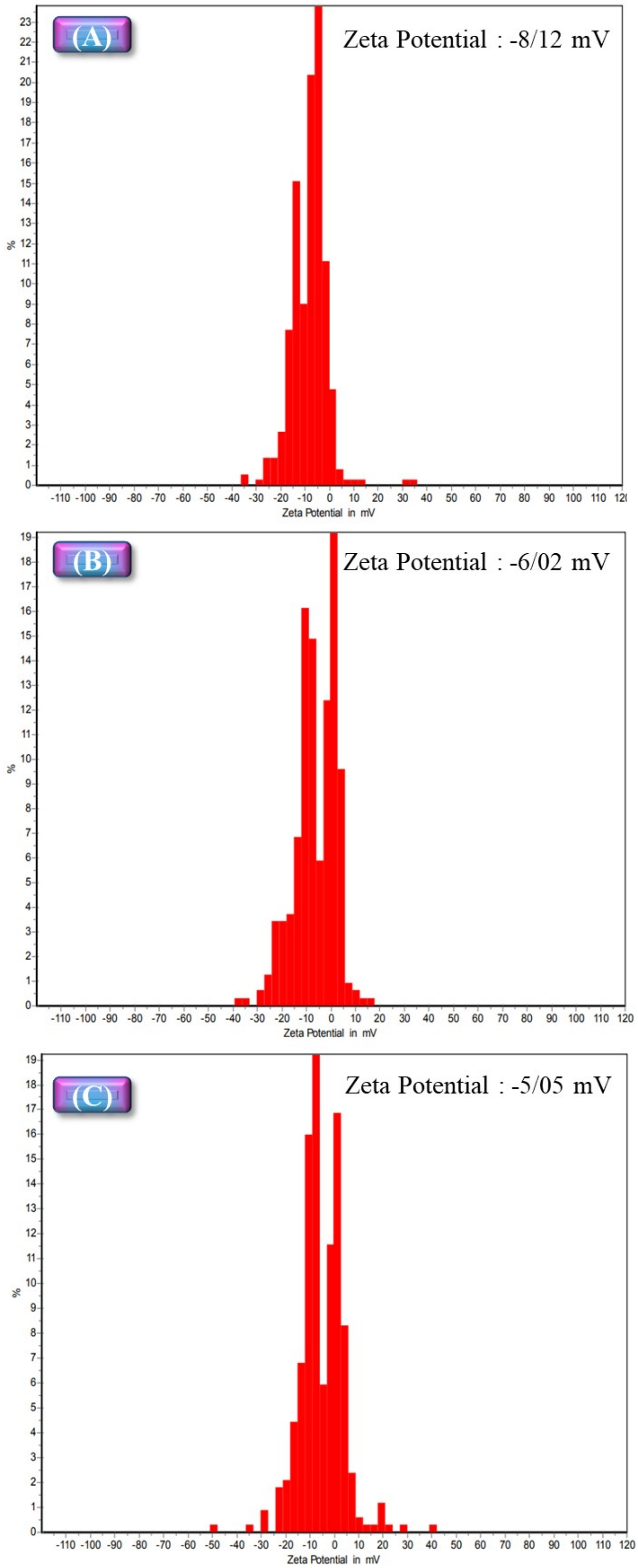

Fig. S3 Zeta potential curves of (A) GHyd, (B) F-GHyd2 and (C) F-GHyd3 formulas.

Table S1. %EE variations by changing the ratio of F-GHyd2 to DNP (mean ±SD, n=3).

F-GHyd2 to DNP ratio (mg/ml / mg/ml)	*500.00*	*250.00*	*25.00*	*12.50*	*6.25*
%EE	83.12± 2.20	85.65±3.36	95.85± 1.44	93.12± 3.10	87.61± 3.31

FTIR analysis of DNP/hydrogel interactions

Considering the chemical structure of DNP the bands at about 1693 and 1311 cm^{-1} are ascribed to the stretching vibrations of the C=O and C-N groups (Fig. 4A (curve b)). The absorption band around 3297 cm^{-1} in the F-GHyd2 spectra is assigned to the NH2 and OH groups of its structure that overlapped (Fig. 4A (curve a)). The IR spectra of the physical mixture sample (Fig. 4A (curve c)) demonstrate a simple combination of characteristic absorption bands of DNP and F-GHyd2, illustrating only a mixture of the two. Simultaneously, in the complexation of the DNP with the F-GHyd2 hydrogel spectrum (Fig. 4A (curve d)), the characteristic bands at 1693 and 1311 cm^{-1} declined. Also, the observed shift in the characteristic broad peak of the NH2 and OH groups (3369 cm^{-1}) suggested some interactions between DNP and F-GHyd2. It can be pointed out that, due to the remaining functional groups and bands of donepezil in the spectrum d, there were no chemical interactions between the F-GHyd2 and DNP.

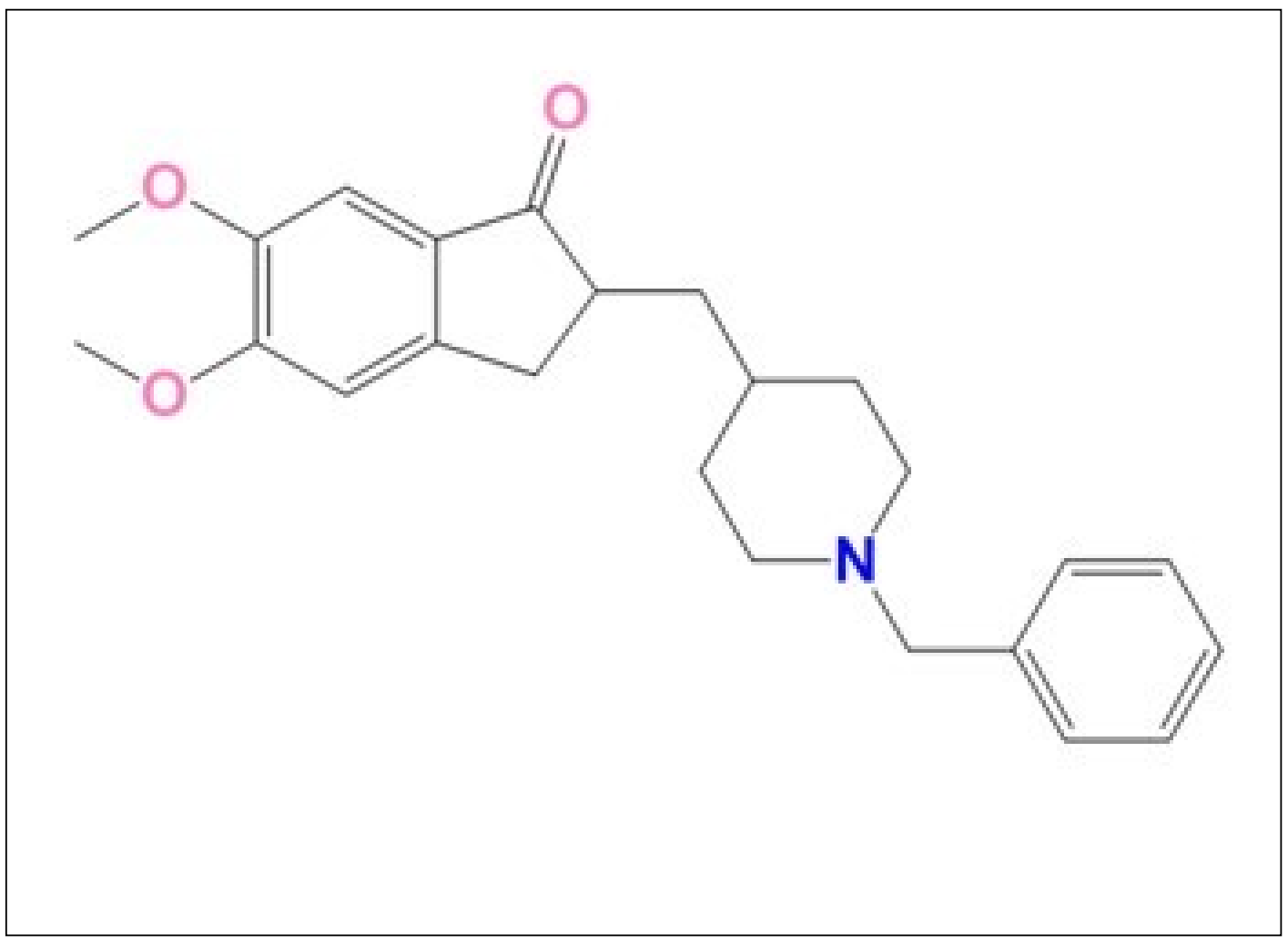

Fig. S4 Chemical structure of Donepezil (DNP).

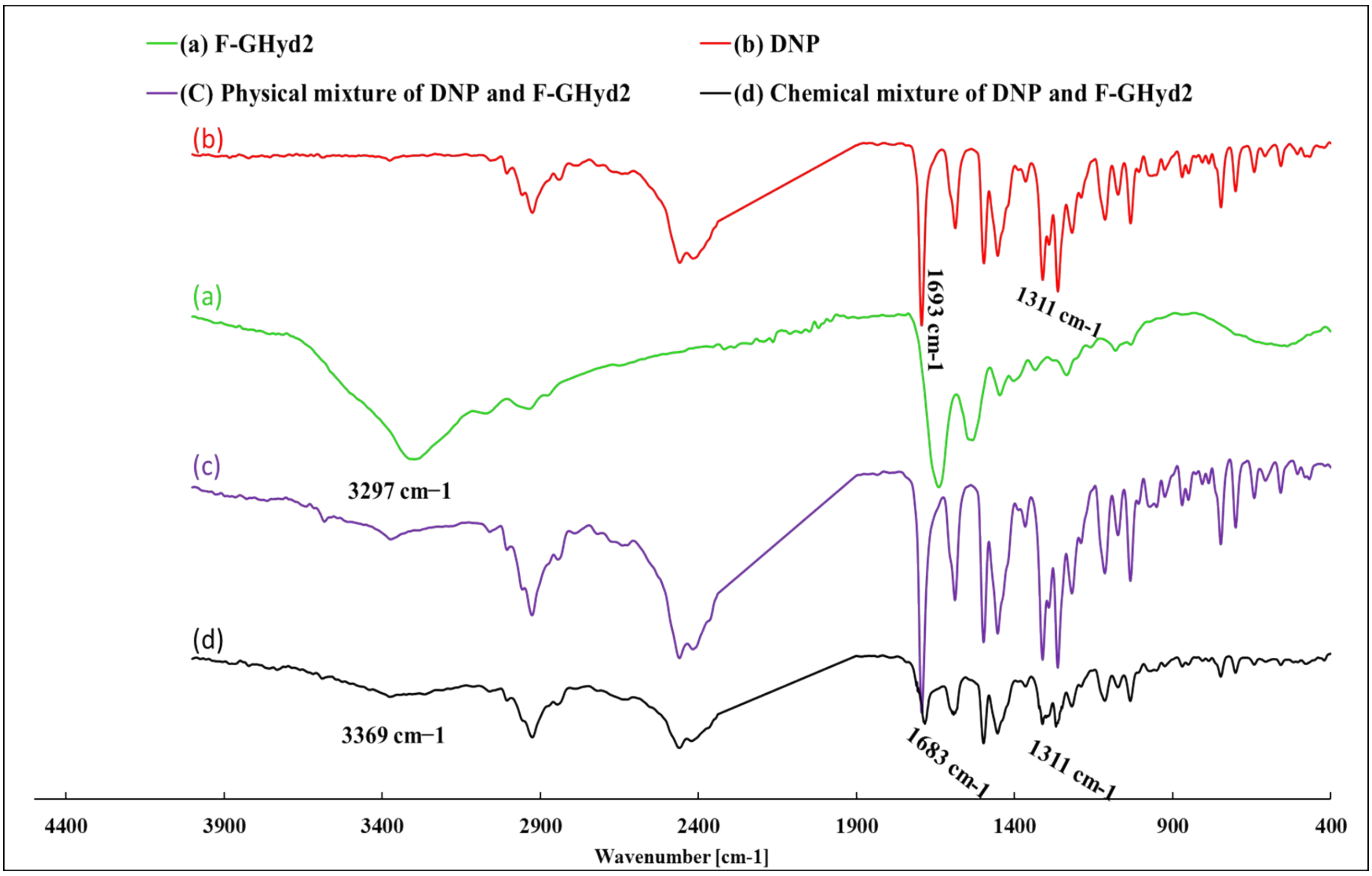

Fig. S5 FTIR spectra of (a) F-GHyd2, (b) DNP, (c) physical mixture, and (d) chemical mixture of the DNP with F-GHyd2 (DNP/F-GHyd2)

TGA analysis

The thermographs of hydrogels (GHyd, F-GHyd2, and F-GHyd3) are shown in Fig. S6. The thermal degradation process of hydrogels consists of two steps, and the initial peak is attributed to the evaporation of the trapped water molecules related to the presence of the hydrophilic functional groups (-OH, -C-O-C- and –NH2) of the prepared hydrogels [50]. The main degradation of GHyd with a maximum weight loss happening at 322 °C corresponds to the rupture of the peptide bonds and polypeptide chains and a weight loss of nearly 41.6% are observed. Incorporating the 10 % SPM-β-CD polyamine into the hydrogel network (F-GHyd2) resulted in a weight loss of about 38.8% at 325 ^{o}C. Although the amount of weight loss was decreased compared to the GHyd, it didn't change remarkably in the TGA curve. This could be due to the destruction of the hydrogen bond and crystalline structure of β-CD with attachment to the gelatin.

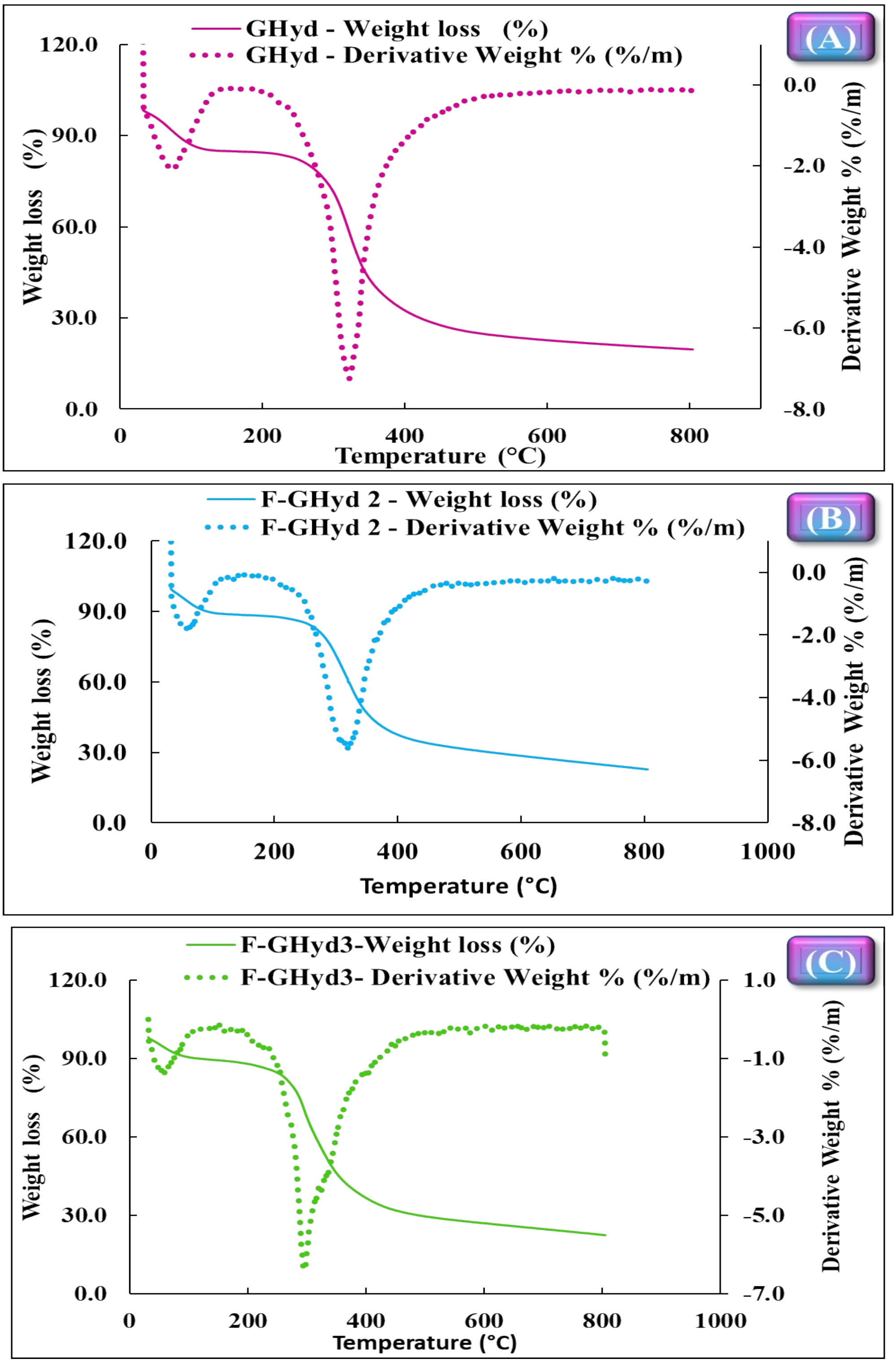

Fig. S6 TGA and derivative thermogravimetry (DTG) curves of (A) GHyd, (B) F-GHyd2 and (C) F-GHyd3. [stick lines display the TGA thermogram that represents the weight loss for each

hydrogel as a function of temperature, and dashed lines illustrate the DTG curve representing the weight loss rate for each hydrogel against time].

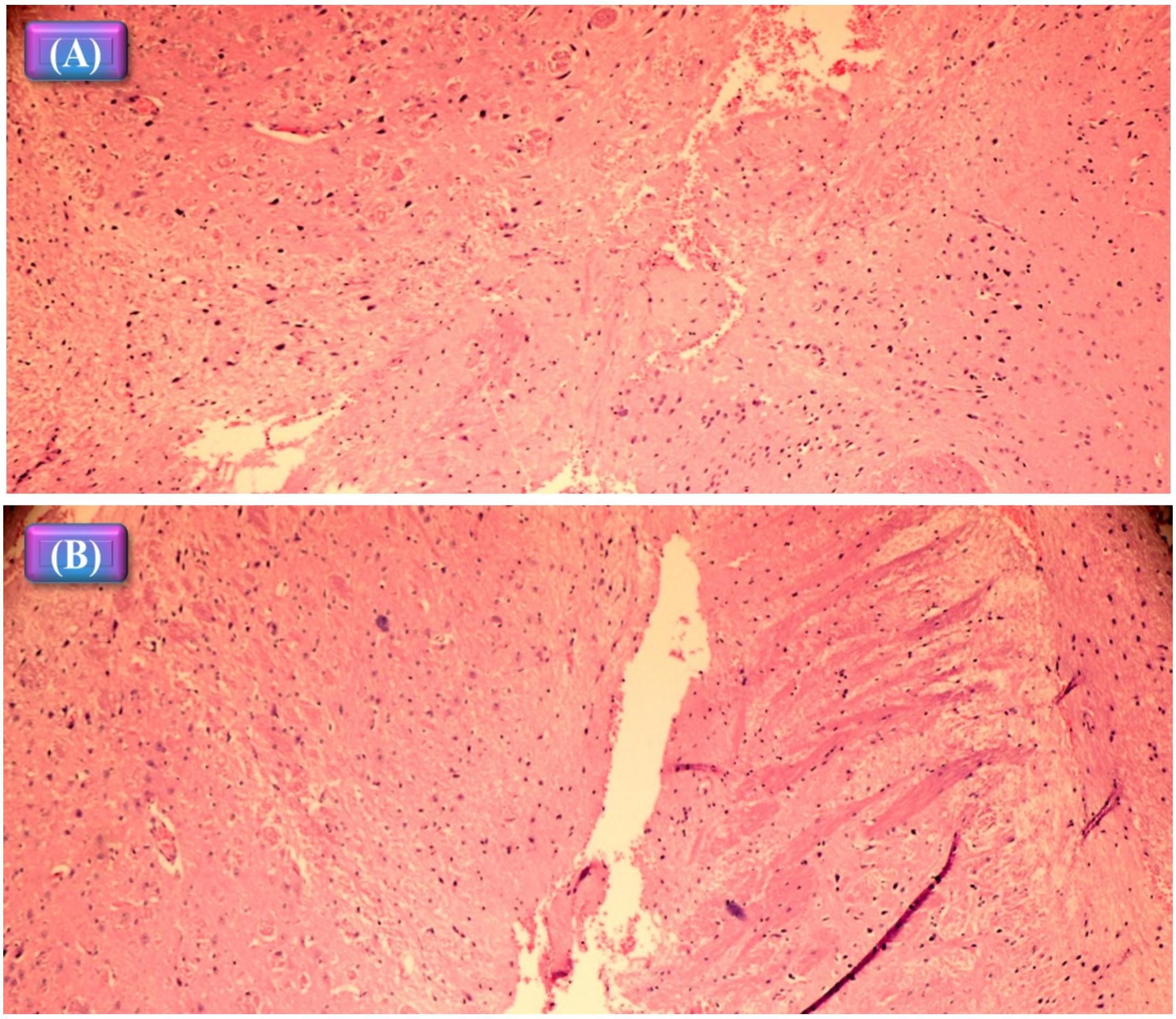

Fig. S7. Histopathological photomicrograph of the rats' brain after administering (A) the free DNP solution and (B) DNP/F-GHyd2.

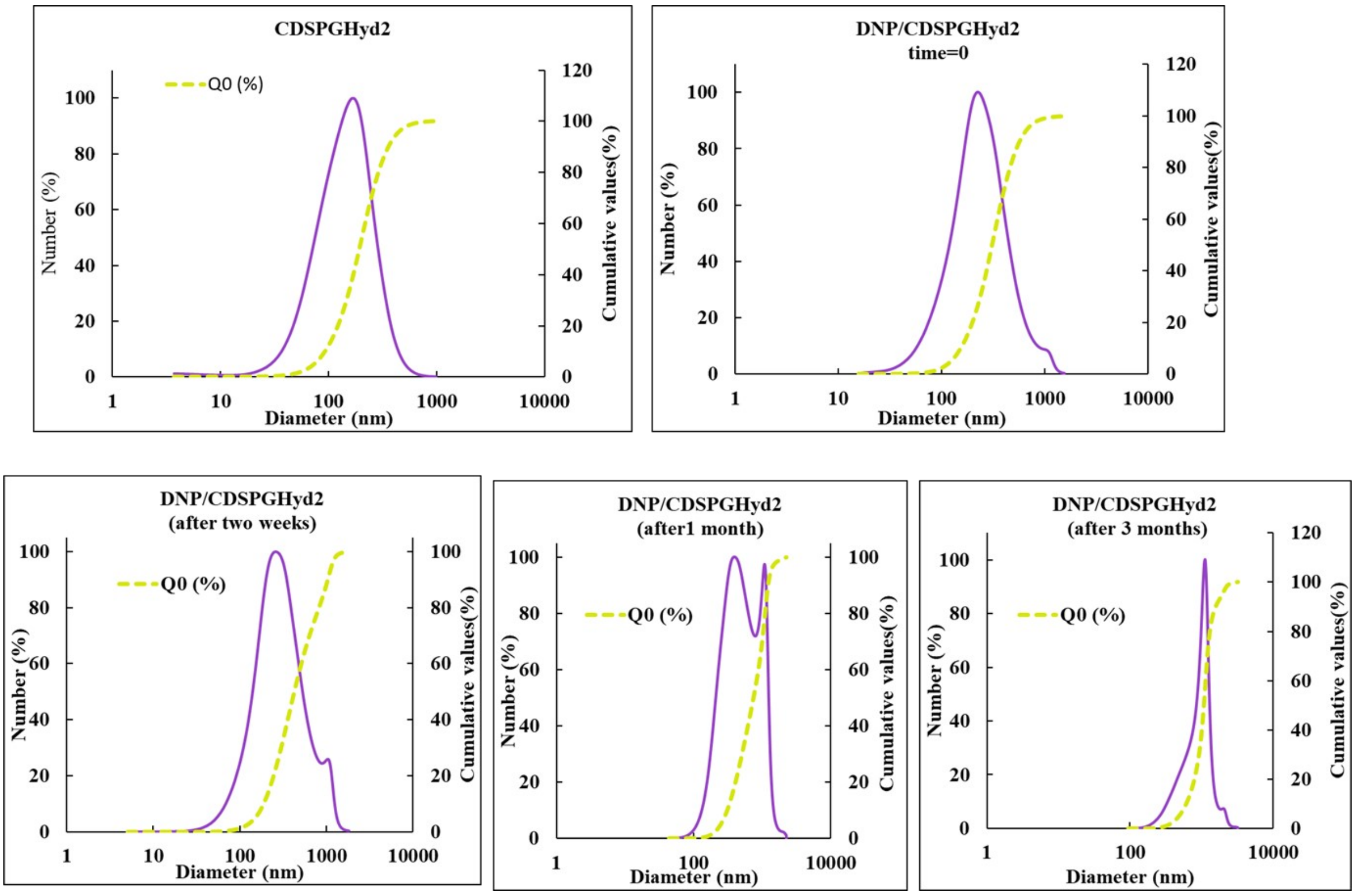

Fig. S8 DLS results of F-GHyd2 and DNP/ F-GHyd2 at time=0, after two weeks, one month and three months [straight lines show the Numbers and dashed lines show the Cumulative values].

Table S2. DLS results and %radical scavenging effect of F-GHyd2 and DNP/ F-GHyd2 in time=0 and after two weeks, one month, and three months (mean ±SD, n=3).

Samples	*Size (nm)*	*Cumulative Value (%)*	*Scavenging Percent (±%RSD)*
F-GHyd2	175	69% (< 246 nm)	40.29 ± 3.06
DNP/F-GHyd2	217	69% (< 372 nm)	72.33 ± 3.43
DNP/F-GHyd2 (after two weeks)	261	58% (< 499 nm)	70.20 ± 1.22
DNP/F-GHyd2 after one (month)	422-1066	22% (< 422 nm)<br>84% (< 1113 nm)	69.95 ± 2.29
DNP/F-GHyd2 (after three months)	1115	67% (< 1178 nm)	68.26 ± 4.01

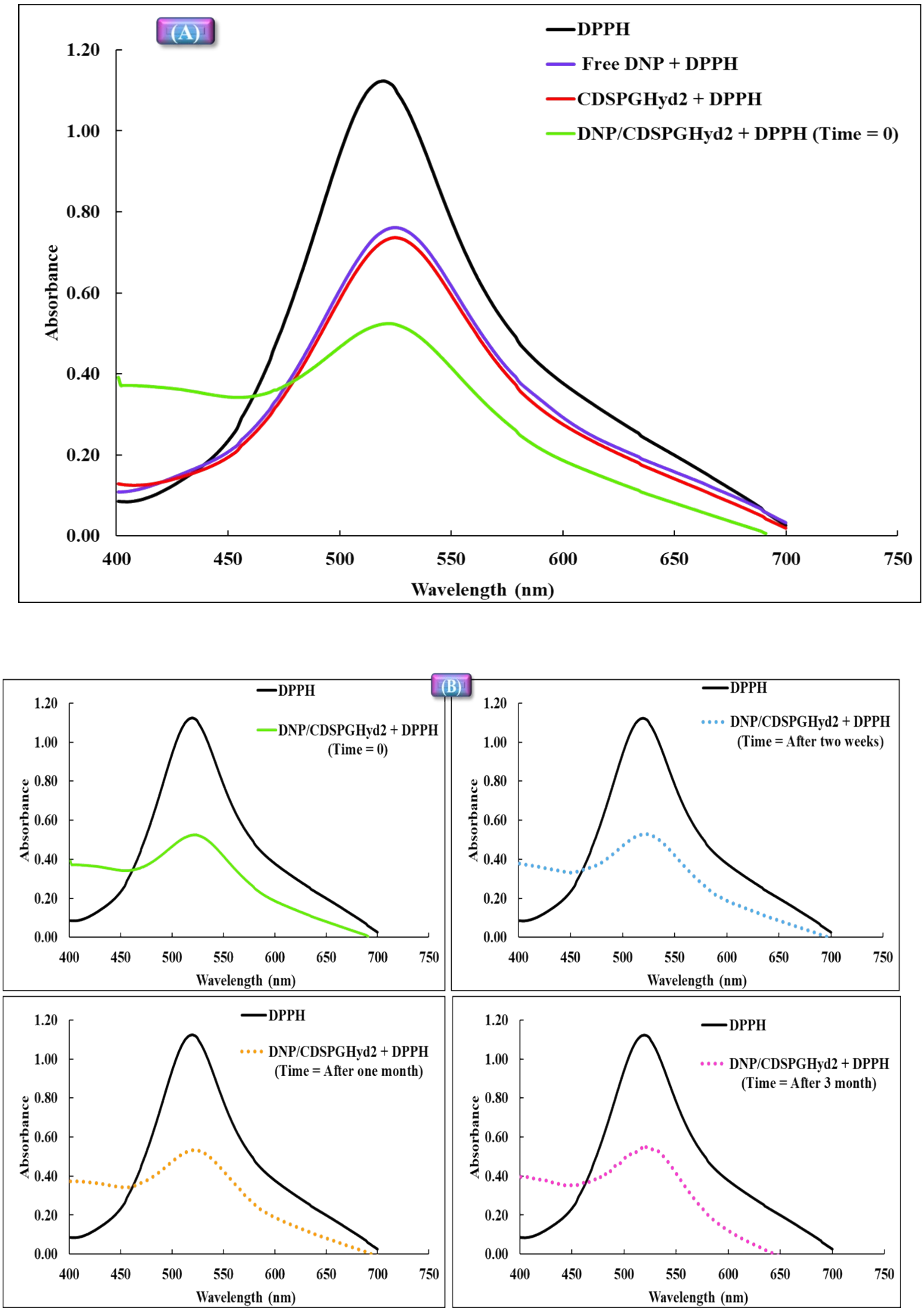

Fig. S9 Investigation of the radical scavenging of samples by DPPH assay, (A) UV/Visible spectrum DPPH in the presence of F-GHyd2 and DNP/F-GHyd2 at time=0 and (B) UV/Visible

spectrum DPPH in the presence of samples at time=0, after two weeks, one month and three months.
